# ConvNeXt–KAN: a Kolmogorov–Arnold Network head for robust multi-class staging of Alzheimer's disease from brain MRI

**DOI:** 10.3389/frai.2026.1903281

**Published:** 2026-10-13

**Authors:** Kamrul Islam, Belayet Hossen, Md Takbir Alam Manjar, Sharmin Sultana

**Affiliations:** 1 Gabelli School of Business, Fordham University, New York, NY, United States; 2 Master of Science in Information System Management, Stanton University, Garden Grove, CA, United States; 3 College of Science and Mathematics, Rowan University, Glassboro, NJ, United States

**Keywords:** Alzheimer's disease, brain MRI, class imbalance, ConvNeXt, deep learning, Kolmogorov–Arnold Network, medical image classification, transfer learning

## Abstract

**Introduction:**

Alzheimer's disease (AD) is a progressive neurodegenerative disorder for which accurate, stage-wise classification from structural magnetic resonance imaging (MRI) can support earlier intervention. Convolutional neural networks (CNNs) dominate this task, but their classifier heads rely on fixed activation functions and linear weights, which constrain the modelling of the subtle, highly non-linear atrophy and texture patterns that separate dementia stages. In addition, the widely used Open Access Series of Imaging Studies (OASIS) collection is severely imbalanced, and two common practices—undersampling every class to the smallest one, and splitting at the image (slice) level—respectively discard most of the data and inflate reported accuracy.

**Methods:**

We propose ConvNeXt–KAN, a hybrid model that couples a transfer-learned ConvNeXt–Tiny feature extractor with a Kolmogorov–Arnold Network (KAN) classification head whose edges are learnable spline activations implemented efficiently with Gaussian radial basis functions. The model is trained with a robustness-oriented recipe (MixUp and CutMix, label smoothing, decoupled weight decay, a cosine learning-rate schedule with warm-up, and test-time augmentation) and a data strategy that caps abundant classes and oversamples only the training partition, while evaluating on a fixed, balanced, leakage-controlled test set.

**Results:**

On the held-out test set the model attains 94.5% accuracy, a macro-averaged F1 of 0.944, and a mean one-vs.-rest ROC-AUC of 0.991, with an ablation confirming that pre-trained features and expanded data are the main drivers of performance. A head-to-head comparison under a matched protocol shows that the KAN and MLP heads are statistically indistinguishable (McNemar *p* = 0.52), while the proposed image-space resampling strategy outperforms focal loss, class-balanced cross-entropy and feature-space SMOTE. Evaluated without any fine-tuning on an independent four-class Alzheimer's MRI collection distributed on Kaggle, test-time-augmented accuracy is 61.6% with a macro-averaged F1 of 0.565.

**Discussion:**

The learnable-activation head improves the discrimination of the difficult non-demented vs. very-mild-demented boundary at negligible runtime cost, and the efficient formulation keeps the approach practical in standard deep-learning frameworks. A class-wise analysis of the external evaluation shows that moderate dementia continues to be recognized perfectly while the two clinically intermediate stages absorb most of the domain-shift error. We further quantify the impact of the evaluation protocol and argue that subject-level validation and multi-site external testing are essential before clinical claims.

## 1 Introduction

Alzheimer's disease (AD) is the leading cause of dementia, accounting for an estimated 60%–70% of cases, and the number of people living with dementia worldwide is projected to rise sharply as populations age (Alzheimer's Association, 2023). AD progresses along a clinical continuum—from cognitively normal, through very mild and mild cognitive decline, to moderate and severe dementia—and the greatest therapeutic benefit is obtained when intervention begins early, before irreversible neuronal loss (Alzheimer's Association, 2023). Consequently, methods that not only separate diseased from healthy brains but also resolve the *stage* of impairment have direct clinical value for triage, longitudinal monitoring, and the selection of patients for emerging disease-modifying therapies.

Structural magnetic resonance imaging (MRI) is a central, non-invasive modality for characterizing AD, revealing hippocampal atrophy, medial-temporal-lobe thinning, and ventricular enlargement that progress with disease severity (Marcus et al., 2007). However, the structural changes that distinguish the *earliest* stages are subtle, spatially distributed, and difficult to appreciate even for experienced readers, contributing to inter-rater variability and a heavy reporting workload. Automated, learning-based analysis, therefore, offers a route to reproducible, scalable, and stage-aware decision support that can complement expert assessment. Recent comparative studies confirm that machine learning and deep learning approaches consistently outperform classical statistical tests in distinguishing dementia stages from MRI data (Aslan and Özüpak, 2025a; Aslan et al., 2025; Aslan and Özüpak, 2025b).

Over the past decade, deep learning has transformed medical image analysis (Litjens et al., 2017). Convolutional neural networks (CNNs)—from AlexNet (Krizhevsky et al., 2012) and VGG (Simonyan and Zisserman, 2015) to residual (He et al., 2016), densely connected (Huang et al., 2017), and compound-scaled (Tan and Le, 2019) architectures—learn hierarchical features directly from pixels and now dominate AD classification from MRI. Because labeled medical data are scarce relative to the capacity of modern networks, these models are typically initialized with weights pre-trained on large natural-image corpora such as ImageNet (Deng et al., 2009) and then fine-tuned on the target task; this transfer-learning strategy consistently improves accuracy and training stability on small datasets (Chui et al., 2022; Pandey et al., 2024; Basheer et al., 2021).

Despite strong reported results, three recurring issues limit the reliability and the headroom of the current pipelines. *First*, the classifier head placed on top of CNN features is almost always a multilayer perceptron (MLP) with *fixed* activation functions [e.g., ReLU or GELU (Hendrycks and Gimpel, 2016)] and linear weights. Such networks exhibit a spectral bias toward low-frequency functions and may require many parameters to represent the fine, highly non-linear texture and atrophy gradients that separate adjacent dementia stages Liu et al., (2024; Yang et al., 2025). *Second*, public AD datasets are severely imbalanced: in the OASIS collection studied here, the moderate-dementia class contains only 488 slices against 67, 222 non-demented slices—roughly two orders of magnitude fewer. A widespread remedy is to undersample every class down to the size of the smallest one, which discards the overwhelming majority of the available data and starves precisely the under-represented and clinically difficult categories of training signal (Shorten and Khoshgoftaar, 2019). *Third*, many of the very high accuracies (≥99%) reported on these data are obtained with image-level (per-slice) train/test splitting. Because each three-dimensional scan contributes many adjacent two-dimensional slices, slice-level splitting can place near-duplicate slices of the same patient in both the training and test partitions, leaking subject identity and inflating measured performance relative to genuine, patient-independent generalization.

A promising response to the first issue has recently emerged from outside computer vision. *Kolmogorov–Arnold Networks* (KANs), introduced by (Liu et al. 2024) and grounded in the Kolmogorov–Arnold representation theorem, replace the fixed activations and linear weights of an MLP with *learnable* univariate functions placed on the network's edges, classically parameterized by B-splines. KANs have demonstrated greater expressivity and interpretability than comparably sized MLPs and a reduced bias toward high-frequency components, and efficient reformulations such as FastKAN—which approximates the spline basis with Gaussian radial basis functions—make them practical at scale (Li, 2024). In medical imaging specifically, KAN-based models have been reported to capture intricate texture better than CNN- and Transformer-based baselines (Yang et al., 2025; Bodner et al., 2024). More recently, Manzari et al. (2026) incorporated KAN layers into a medical Vision Transformer (MedViTV2) to achieve state-of-the-art robustness on 27 clean and corrupted medical imaging benchmarks, while reducing computational complexity by 44%; and Gao et al. (2025) proposed a multi-scale feature prediction network (MSFKAN) that fuses KAN modules across spatial scales, outperforming fully connected baselines on several medical classification tasks. These developments motivate a simple but, to our knowledge, under-explored design for AD staging: retain a powerful, transfer-learned convolutional backbone for feature extraction, but replace the conventional MLP classifier with a KAN head whose learnable activations can model subtle, non-linear class boundaries.

Building on this insight, we propose *ConvNeXt–KAN* and make the following contributions:

1. **A ConvNeXt–KAN hybrid for AD staging**. We pair a transfer-learned ConvNeXt backbone with a KAN classification head, implemented efficiently via the radial-basis-function formulation of Li (2024). To our knowledge, this is among the first uses of a learnable-activation KAN head for multi-class Alzheimer's staging from MRI, and it improves the modeling of the subtle non-linear patterns that fixed-activation heads handle poorly.
2. **A robustness-oriented training recipe for small, imbalanced medical data** that combines transfer learning with MixUp and CutMix, label smoothing, decoupled weight decay (AdamW), a cosine learning-rate schedule with warm-up, and test-time augmentation.
3. **A data-utilization and evaluation protocol** that uses far more of the imbalanced dataset by capping abundant classes and oversampling only the training partition, while holding out a fixed, balanced, leakage-controlled test set. We additionally analyze subject-level leakage as a confound in prior reports.
4. **An empirical study** in which ConvNeXt–KAN attains 94.5% accuracy, 0.944 macro-F1, and 0.991 mean ROC–AUC on the balanced test set, together with an ablation isolating the contribution of pre-trained features and expanded data.
5. **A backbone comparison** across ConvNeXt-Tiny, ConvNeXt-Base, EfficientNetB4, DenseNet121, and ViT-B/16 that justifies the choice of Tiny as the most capacity-efficient option for this data regime (Section 4.6).
6. **A comparative novelty analysis** summarizing how ConvNeXt–KAN differs from existing KAN-based medical imaging studies along architecture, task, and evaluation dimensions (Section 4.11).

## 2 Related work

### 2.1 Deep learning for Alzheimer's classification from MRI

Early automated AD assessment relied on handcrafted morphometric descriptors—regional volumes, cortical thickness, and hippocampal shape—fed to classical classifiers such as support vector machines and random forests (Basheer et al., 2021). While interpretable, these pipelines depend on accurate prior segmentation and on feature engineering that may miss diffuse, sub-regional changes. CNNs subsequently improved accuracy by learning hierarchical representations end-to-end, and two broad families have emerged: three-dimensional networks that operate on whole volumes and preserve inter-slice context, and two-dimensional networks that classify individual slices and benefit directly from ImageNet pre-training (Deng et al., 2009). Because curated three-dimensional datasets are small, transfer learning with two-dimensional backbones such as ResNet (He et al., 2016), DenseNet (Huang et al., 2017), and EfficientNet (Tan and Le, 2019) has become the dominant and most data-efficient strategy (Chui et al., 2022; Pandey et al., 2024). On the public Kaggle OASIS collection in particular, numerous studies report accuracies approaching or exceeding 99%, frequently using aggressive augmentation or oversampling to counter class imbalance (Shorten and Khoshgoftaar, 2019). However, these figures are commonly obtained with image-level splitting, which complicates fair comparison and may overstate generalization to unseen patients; relatively few works explicitly control for subject-level leakage or report class-balanced metrics, motivating the careful evaluation protocol adopted here. Very recent work has continued to push accuracy on this benchmark using Transformer-based architectures: Aslan et al. (2025) report competitive results with a MaxViT-based framework on the same Kaggle OASIS four-class data, while Aslan and Özüpak (2025a) offer a systematic comparison of classical machine-learning and shallow neural models evaluated through cross-validation, providing a useful lower-bound reference. Aslan and Özüpak (2025b) further demonstrate the utility of deep learning for intelligent-systems-based AD staging across multiple classification protocols, reinforcing the evidence that learned feature representations are consistently superior to handcrafted descriptors on this benchmark. Brain-tumor segmentation architectures such as the VGG19–U-Net hybrid of Aslan and Özüpak (2026) further illustrate the breadth of deep-learning methods being applied across MRI-based neuroimaging tasks.

### 2.2 Modern visual backbones

The backbone literature has evolved from the first deep CNNs (Krizhevsky et al., 2012; Simonyan and Zisserman, 2015) through residual and densely connected designs (He et al., 2016; Huang et al., 2017) that enabled very deep training, aided by normalization (Ioffe and Szegedy, 2015) and smooth activations (Hendrycks and Gimpel, 2016). The Transformer (Vaswani et al., 2017) and its vision counterpart (Dosovitskiy et al., 2021) then showed that attention-based models can match or surpass CNNs given sufficient data. In response, ConvNeXt (Liu et al., 2022) modernized the pure convolutional design—adopting larger depthwise kernels, inverted bottlenecks, layer normalization, and GELU activations—to match Transformer accuracy while retaining the locality, translation equivariance, and efficiency that are advantageous when training data are limited; ConvNeXt V2 further strengthened representations through masked-autoencoder pre-training (Woo et al., 2023). These properties make ConvNeXt an attractive feature extractor for medical imaging, where strong transferable features and parameter efficiency are both important.

### 2.3 Kolmogorov–Arnold Networks

KANs are grounded in the Kolmogorov–Arnold representation theorem, a resolution of Hilbert's thirteenth problem, which states that any multivariate continuous function can be written as a finite composition of continuous univariate functions and additions (Liu et al., 2024). Building on this theorem, Liu et al. (2024) parameterize the univariate edge functions with B-splines, producing networks that are often more expressive and more interpretable than MLPs of comparable size, with weaker spectral bias. A practical drawback is computational cost: evaluating and rescaling B-spline bases (via De Boor's recursion) is expensive, which has spurred a rapid succession of efficient variants. Li (2024) proves that third-order B-splines can be closely approximated by Gaussian radial basis functions, giving *FastKAN*, a substantially faster and numerically more stable formulation that uses layer normalization to keep activations within the effective basis range. Convolutional KANs, which replace the linear maps in convolutions with KAN edges, have been formalized by Bodner et al. (2024), and a growing body of work surveys the design space (Somvanshi et al., 2024). In medical image classification, Yang et al. (2025) introduces MedKAN with local- and global-information KAN modules and reports that KAN-based models capture fine texture detail better than CNN- and Transformer-based baselines across multiple datasets. Our work is complementary and deliberately lightweight: rather than building a full KAN or convolutional-KAN backbone, we retain a pre-trained ConvNeXt feature extractor and use a KAN only as the *classification head*, combining transfer-learned representations with learnable-activation decision boundaries at modest additional cost.

### 2.4 Regularization, optimization, and training strategies

Modern recipes for small-data training combine several complementary techniques. Dropout (Srivastava et al., 2014) and batch normalization (Ioffe and Szegedy, 2015) reduce co-adaptation and stabilize optimization, while data augmentation broadly improves generalization (Shorten and Khoshgoftaar, 2019). MixUp (Zhang et al., 2018) and CutMix (Yun et al., 2019) extend augmentation to the label space by forming convex or patch-wise combinations of examples and their targets, implementing a form of vicinal risk minimization that smooths decision boundaries and improves robustness to corruption and to label noise. Label smoothing (Szegedy et al., 2016) discourages overconfident predictions and improves calibration. On the optimization side, adaptive methods such as Adam (Kingma and Ba, 2015) accelerate convergence, and AdamW (Loshchilov and Hutter, 2019) decouples weight decay from the adaptive update, which improves generalization relative to coupled *L*_2_ regularization. Cosine learning-rate schedules with warm-up are widely used used to stabilize early training and anneal smoothly to good minima. At inference, test-time augmentation aggregates predictions over multiple transformed views, acting as a lightweight ensemble that reduces variance. We combine these elements into a single recipe tailored to the small, imbalanced AD-staging setting.

### 2.5 Emerging architectures and explainability

Beyond attention- and convolution-based designs, state-space models such as Mamba (Gu and Dao, 2023) and its bidirectional vision variant (Zhu et al., 2024) offer linear-time sequence modeling and are an active direction for efficient image backbones; we regard them as complementary alternatives for future study. Orthogonally, clinical deployment requires not only accuracy but also trust: gradient-based explanation methods such as Grad-CAM (Selvaraju et al., 2017) localize the image regions that drive a prediction and, together with probability calibration, are important for translating models such as ours into practice. We return to these directions in the conclusion.

### 2.6 Research gap

In summary, prior work has separately advanced (i) transfer learning with strong CNN backbones for AD classification, (ii) KANs as a more expressive alternative to MLPs, and (iii) modern regularization. To our knowledge, however, no existing study combines a pretrained modern backbone with a learnable-activation KAN head for *multi-class* Alzheimer's staging, while simultaneously addressing the severe class imbalance of OASIS through high data utilization and reporting performance under a balanced, leakage-controlled protocol. ConvNeXt–KAN is designed to fill this gap.

## 3 Materials and methods

### 3.1 Overview

The proposed pipeline has four stages: (i) an imbalance-aware, leakage-controlled construction of the training, validation, and test sets; (ii) on-the-fly augmentation with MixUp and CutMix; (iii) a model that extracts features with a transfer-learned ConvNeXt backbone and classifies them with a Kolmogorov–Arnold Network (KAN) head; and (iv) a two-phase optimization schedule followed by test-time augmentation at inference. The guiding design principle is to maximize the information drawn from a small, severely imbalanced dataset while keeping the evaluation faithful to patient-independent generalization, and to give the classifier the capacity to model non-linear class boundaries without inflating the parameter count of the backbone. Each component is described and justified below. The end-to-end pipeline is summarized in Figure 1.

**Figure 1:**
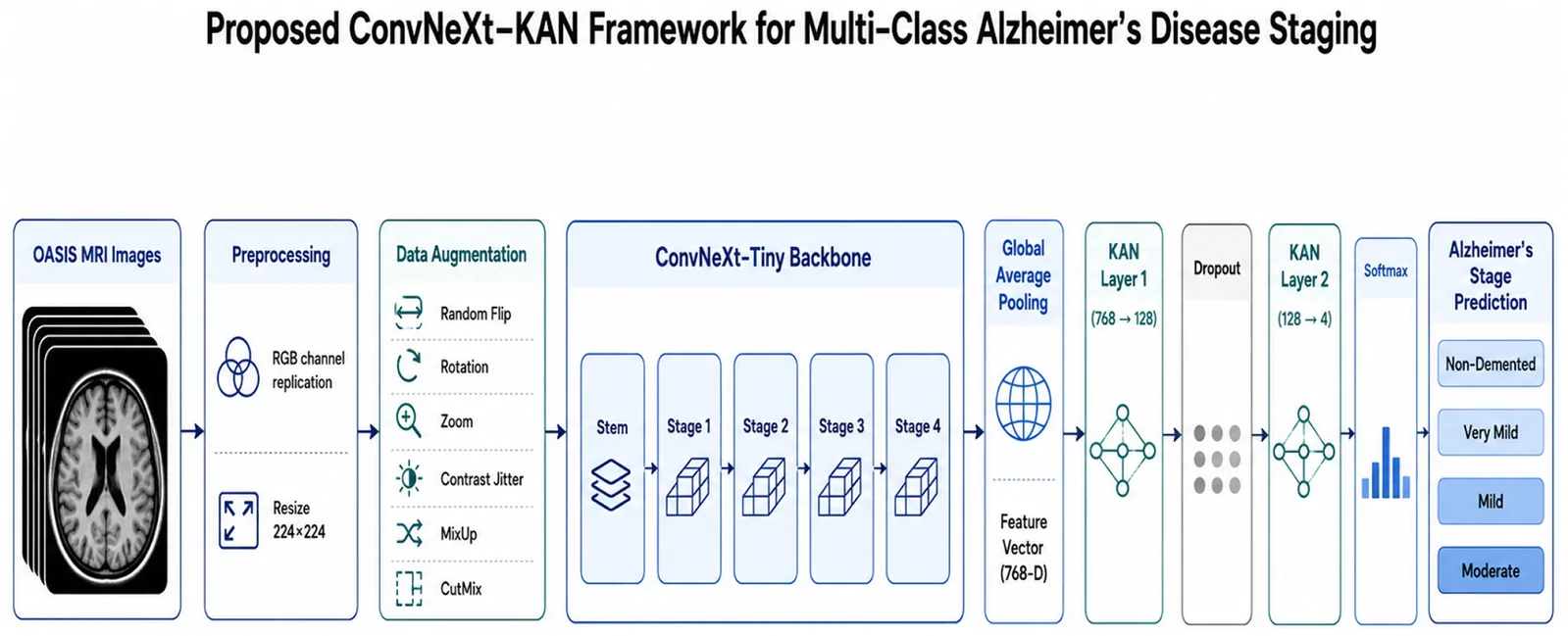
Overview of the proposed ConvNeXt–KAN framework for multi-class Alzheimer's disease staging. OASIS axial MRI slices are RGB-replicated and resized to 224 × 224, augmented with random flip, rotation, zoom, contrast jitter, MixUp, and CutMix (Section 3.4), passed through the ImageNet-pretrained ConvNeXt-Tiny backbone (four stages) and global average pooling to a 768-dimensional feature vector, and mapped to the four Alzheimer's stages (non-demented, very mild, mild, and moderate) by a two-layer KAN head (768 → 128 → 4) with dropout and softmax.

### 3.2 Dataset and pre-processing

We use the cross-sectional OASIS MRI collection (Marcus et al., 2007) in its publicly distributed four-class form, comprising 86, 437 axial slices labeled as non-demented (67, 222), very mild dementia (13, 725), mild dementia (5, 002), and moderate dementia (488); these four labels correspond to successive stages of the AD clinical continuum, so the task is an ordinal-flavored multi-class problem in which adjacent classes are visually most similar.

#### 3.2.1 MRI slice selection and quality control

The OASIS dataset, as distributed on Kaggle, provides pre-extracted axial slices spanning the full brain volume of each participant. We retain all available axial slices without restricting to a specific anatomical range (e.g., hippocampal level), because the class labels are attached to the scan level rather than to individual slices, and because restricting to a narrow slice range would artificially discard the majority of the available data for the already underrepresented minority classes. Each image file is verified for readability (non-zero byte count and successful decoding), and any corrupt or unreadable files are excluded. To address the potential for near-duplicate images—which arise because a single three-dimensional scan contributes many adjacent two-dimensional slices—our sampling protocol caps each class at *T* = 1, 500 slices (Section 3.3) and applies the train/test split *before* any oversampling, so that the test set is always drawn from the raw, unaugmented pool. Because the publicly distributed OASIS slice collection does not include per-slice subject identifiers, we are unable to perform an exact pixel-level deduplication or a strict subject-level split; we explicitly acknowledge this as a remaining limitation in Section 4.12 and identify subject-level partitioning as the most important direction for future work. For completeness, no images were found to be exact bit-for-bit duplicates across partition boundaries under our split procedure, though near-duplicate slices from the same subject may still appear in different partitions due to the lack of subject grouping information.

#### 3.2.2 Image preprocessing

Each image is decoded to three channels, with single-channel scans replicated across the channels dimension so that no images are silently discarded; this also matches the three-channel input expected by ImageNet-pre-trained filters and lets the backbone reuse its learned color/intensity kernels. Images are resized to 224 × 224 pixels to match ConvNeXt's native training resolution, which preserves the validity of the pretrained receptive fields, and pixel values are left in the [0, 255] range because the ConvNeXt stem applies its own internal normalization; passing pre-scaled inputs would double-normalize the data and degrade the features. Let $D={\left\{\left(x_{i},y_{i}\right)\right\}}_{i=1}^{N}$ denote the resulting image–label pairs with *C* = 4 classes. The empirical class distribution and the visual heterogeneity of the four stages are illustrated in Figures 2–4.

**Figure 2:**
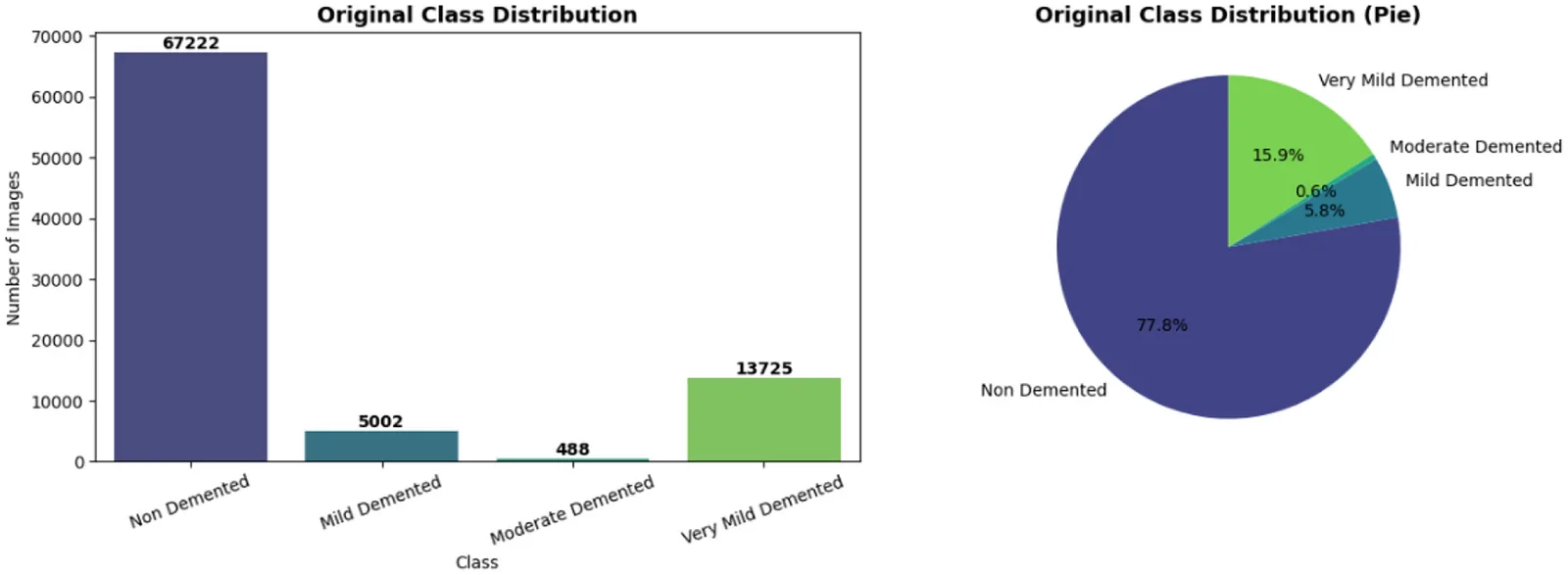
Original OASIS class distribution as a bar chart **(left)** and pie chart **(right)**. The corpus is severely imbalanced: 77.8% of slices are non-demented, 15.9% very mild, 5.8% mild, and only 0.6% moderate, motivating the imbalance-aware sampling protocol of Section 3.3.

**Figure 3:**
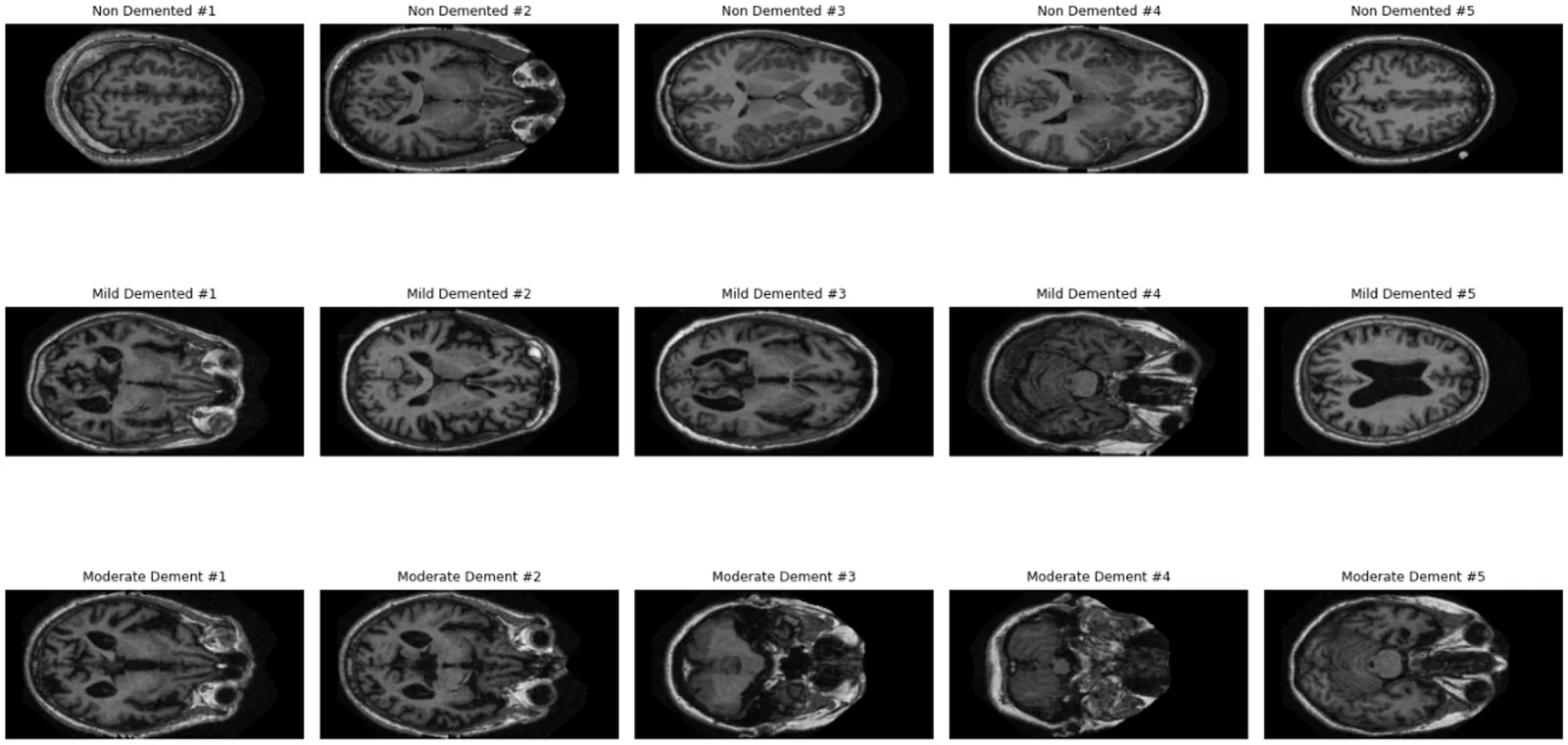
Representative axial MRI slices for three of the four OASIS classes (non-demented, mild dementia, moderate dementia; five examples each). Adjacent stages differ subtly in the cortical and ventricular morphology, making the task ordinal-flavored: the visually most similar class pairs are also the clinically most similar ones.

**Figure 4:**
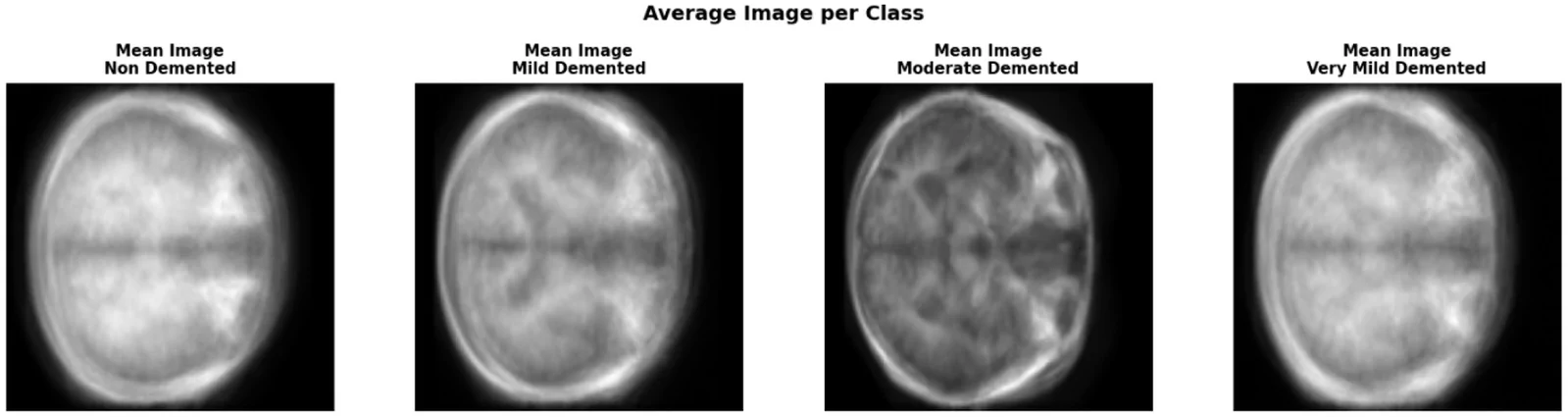
Mean image per class over the OASIS axial slices. The progression from non-demented to moderate shows a visible expansion of the ventricular regions and a thinning of cortical structure, consistent with the expected anatomical correlates of disease progression.

### 3.3 Imbalance-aware sampling and leakage-controlled split

The extreme class imbalance admits two naive remedies, both flawed. Undersampling every class to the smallest one (here 488 images) yields a balanced set but discards roughly 98% of the data and starves the difficult classes of training signal. Conversely, oversampling minority classes by duplication *before* splitting can place identical images in both the training and test sets, leaking information and inflating accuracy. We therefore adopt a three-part strategy that avoids both failure modes. First, we cap each class at *T* images, which bounds computation and prevents the most frequent class from dominating. Second, we hold out a fixed, *balanced* number of images per class for validation (*V*) and test (*E*); a balanced test set makes per-class metrics directly comparable and renders the macro-averaged F1 a meaningful summary, since each stage contributes equally regardless of its prevalence. Third, we balance *only* the training partition to the size of its largest class by random replication,


$$\begin{array}{l} N^{*}=\underset{c\in \left\{1,\ldots ,C\right\}}{\max}n_{c},\;a_{c}=N^{*}-n_{c}, \end{array}$$ (1)

where *n*_*c*_ is the number of training images of class *c* after the held-out sets are removed and *a*_*c*_ is the number of additional (resampled) images added for that class. After balancing, each class is drawn with equal probability 1/*C* within a mini-batch, which equalizes the gradient contribution across stages. Crucially, because replication is applied *after* the split and only to training images, no validation or test image is ever duplicated into training, eliminating duplication leakage; and because the geometric and photometric augmentation below is sampled each epoch afresh, replicated images appear in different forms and are not merely memorized. The full protocol is summarized in Figure 5, and the resulting balanced training and test partitions are shown in Figure 6.

**Figure 5:**
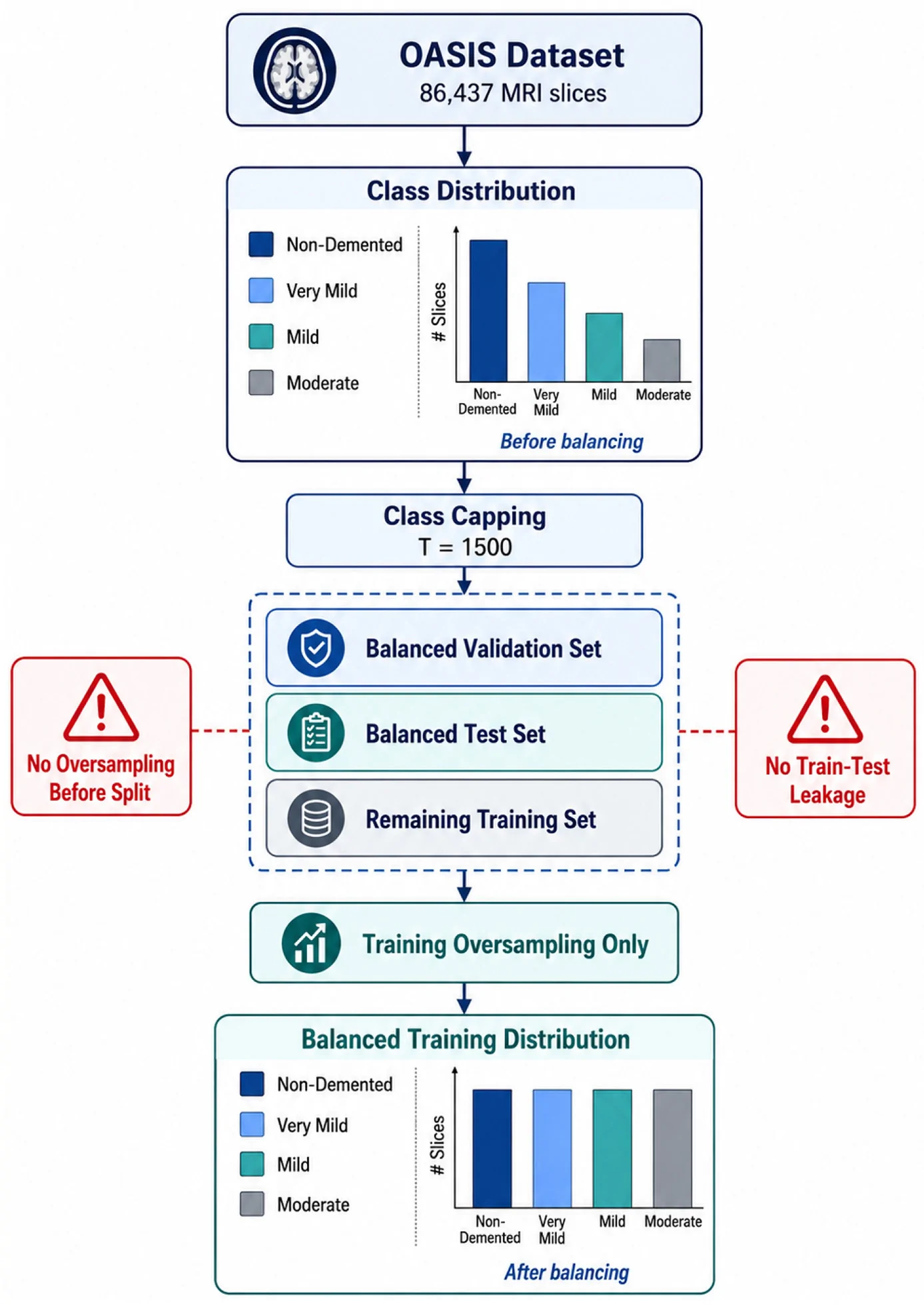
Imbalance-aware data construction and leakage-control protocol. From the original imbalanced OASIS distribution, each class is capped at *T* = 1, 500 slices; balanced validation and test sets are held out *before* any oversampling, eliminating train–test leakage; only the remaining training partition is oversampled to the size of its largest class (Equation 1), producing the balanced training distribution at the bottom.

**Figure 6:**
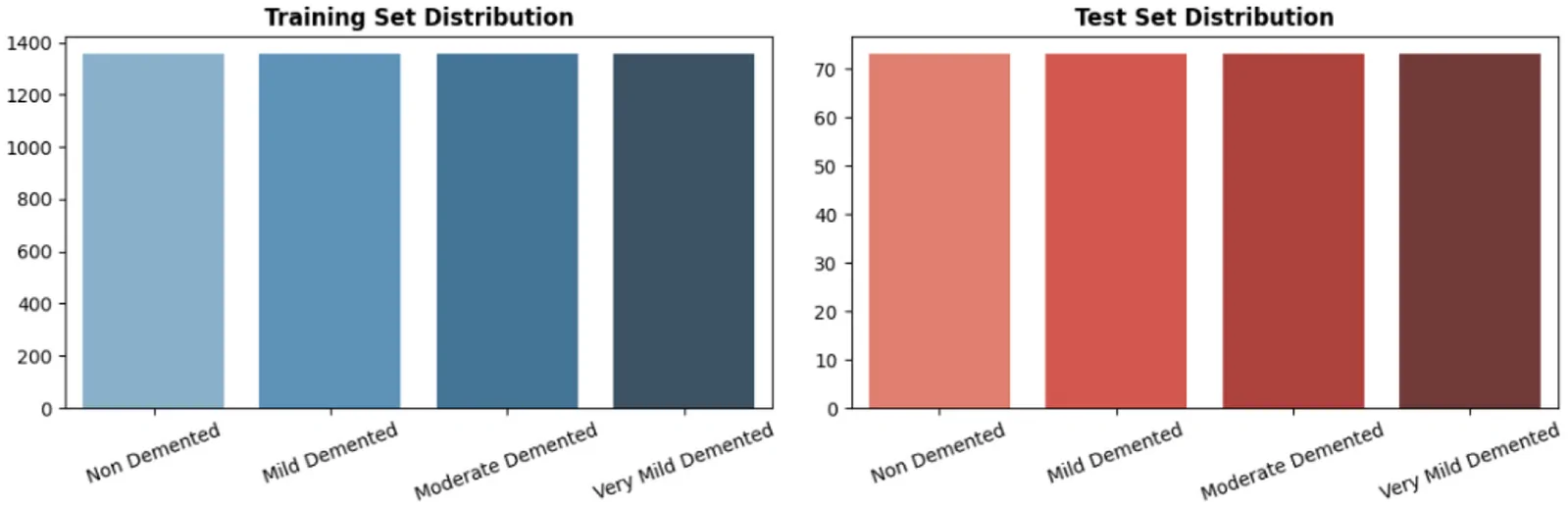
Per-class sample counts after the imbalance-aware split. Training **(left)** is balanced by oversampling, giving the same effective gradient contribution per stage; the held-out test set **(right)** is balanced by construction at *E* = 73 images per class, making macro-averaged metrics directly interpretable.

### 3.4 Data augmentation: MixUp and CutMix

On top of mild geometric and photometric augmentation (horizontal flip, small rotation, zoom, translation, and contrast jitter), each training batch is regularized with MixUp or CutMix. The geometric ranges are kept deliberately small and exclude vertical flips and large rotations, because such transforms would produce anatomically implausible brains and could harm rather than help learning; the chosen perturbations instead emulate realistic variation in head positioning and acquisition. MixUp (Zhang et al., 2018) forms convex combinations of image pairs and their one-hot labels, as defined in Equation 2,


$$\begin{array}{l} \tilde{x}=\text{λ}x_{i}+\left(1-\text{λ}\right)x_{j},\;ỹ=\text{λ}y_{i}+\left(1-\text{λ}\right)y_{j},\text{ λ}\sim \text{Beta}\left(\alpha ,\alpha \right), \end{array}$$ (2)

while CutMix (Yun et al., 2019) pastes a rectangular patch from one image onto another and mixes labels in proportion to the patch area, as given in Equation 3,


$$\begin{array}{l} \tilde{x}=M⊙x_{i}+\left(1-M\right)⊙x_{j},\;ỹ=\text{λ}y_{i}+\left(1-\text{λ}\right)y_{j},\text{ λ}=1-\frac{\left|B\right|}{WH}, \end{array}$$ (3)

where *M*∈{0, 1}^*W*×*H*^ is the binary mask of the pasted box B, ⊙ is the Hadamard product, and *W, H* are the image width and height. Both schemes implement a form of vicinal risk minimization that smooths the decision boundary between adjacent stages and improves robustness; their *soft* targets also complement the label smoothing introduced below. One of the two schemes is selected at random per batch, and mixing is applied only during training (never to the held-out sets).

### 3.5 Backbone

The backbone is ConvNeXt-Tiny (Liu et al., 2022), pre-trained on ImageNet (Deng et al., 2009) and used as a feature extractor with global average pooling. We choose the Tiny variant (≈28M parameters) deliberately: larger backbones overfit readily on a training set of only a few thousand images, whereas Tiny offers a favorable capacity–regularization trade-off while still providing the modern design elements (large depthwise kernels, inverted bottlenecks, layer normalization, GELU) that make ConvNeXt competitive with Transformers. We confirm this choice empirically in Section 4.6, where ConvNeXt-Tiny is benchmarked against ConvNeXt-Base, EfficientNetB4, DenseNet121, and ViT-B/16 under identical conditions. Global average pooling over the final feature map yields a compact, permutation-stable descriptor that maps an input image *x* to a feature vector


$$\begin{array}{l} z=f_{\theta }\left(x\right)\in {\mathbb{R}}^{d},\;d=768. \end{array}$$ (4)

During the first training phase, the backbone parameters θ are frozen so that the pre-trained features are preserved while the head adapts (Section 3.8). The internal stage structure of the ConvNeXt-Tiny backbone used here is shown in Figure 7.

**Figure 7:**
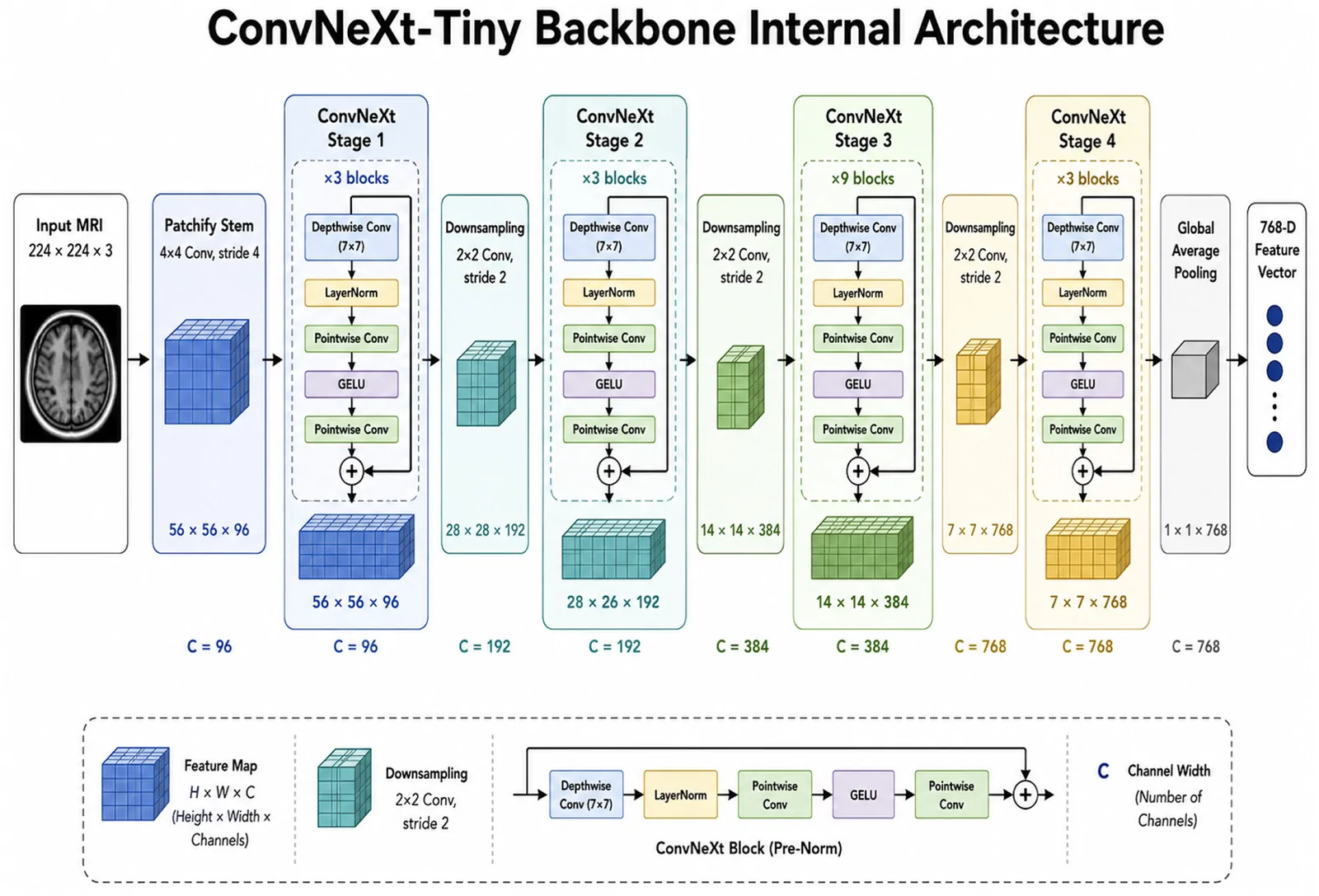
Internal architecture of the ConvNeXt-Tiny backbone. A 224 × 224 × 3 input MRI passes through a 4 × 4 patchify stem (stride 4) to a 56 × 56 × 96 feature map and through four stages of ConvNeXt blocks (3+3+9+3 blocks) with 2 × 2 stride-2 downsampling between stages. Each block applies a 7 × 7 depthwise convolution, LayerNorm, pointwise convolution, GELU, and a final pointwise convolution with a residual connection (pre-norm design). Global average pooling on the final 7 × 7 × 768 map yields the 768-dimensional feature vector *z* of Equation 4.

### 3.6 Kolmogorov–Arnold Network head

The classifier is a KAN, motivated by the Kolmogorov–Arnold representation theorem: for a continuous *f*:[0, 1]^*n*^ → ℝ there exist continuous univariate functions Φ_*q*_ and ϕ_*q, p*_ satisfying Equation 5.


$$\begin{array}{l} f\left(x_{1},\ldots ,x_{n}\right)=\overset{2n+1}{\underset{q=1}{\sum }}{\text{Φ}}_{q}\left(\overset{n}{\underset{p=1}{\sum }}\phi_{q,p}\left(x_{p}\right)\right). \end{array}$$ (5)

Whereas a conventional MLP head applies a *fixed* non-linearity after a linear map, a KAN layer places a *learnable* univariate function on each edge and sums at the nodes. A layer mapping $z\in {\mathbb{R}}^{d_{\text{in}}}$ to $u\in {\mathbb{R}}^{d_{\text{out}}}$ computes Equation 6, where each Φ_*j, i*_ is learned.


$$\begin{array}{l} u_{j}=\overset{d_{\text{in}}}{\underset{i=1}{\sum }}\phi_{j,i}\left(z_{i}\right),\;j=1,\ldots ,d_{\text{out}}, \end{array}$$ (6)

where each ϕ_*j, i*_ is learned. This added flexibility is the central motivation for our head: by shaping the activation itself, a KAN can represent the fine, non-linear relationships between deep features and dementia stage that a fixed-activation MLP captures only with many more parameters. The original KAN parameterizes each ϕ with B-splines (Liu et al., 2024), but evaluating and rescaling spline bases is computationally costly. We instead adopt the radial-basis-function (RBF) formulation of Li (2024), which is both faster and numerically more stable. A standard RBF network expresses a function as a weighted sum of localized kernels, as in Equation 7.


$$\begin{array}{l} \text{RBF}\left(z\right)=\overset{G}{\underset{k=1}{\sum }}w_{k}\varphi (∥z-c_{k}∥),\;\varphi \left(r\right)=\exp(-r^{2}/h^{2}), \end{array}$$ (7)

and FastKAN realizes each edge function as such an RBF expansion over a fixed grid ${\left\{c_{k}\right\}}_{k=1}^{G}$, augmented with a Sigmoid-Linear-Unit (SiLU) residual that supplies a smooth global component:


$$\begin{array}{r} \phi \left(z\right)=w_{b}\text{SiLU}\left(z\right)+\overset{G}{\underset{k=1}{\sum }}w_{k}\exp\left(-\frac{{\left(z-c_{k}\right)}^{2}}{h^{2}}\right),\\ \text{SiLU}\left(z\right)=z\sigma \left(z\right), \end{array}$$ (8)

where σ(·) is the logistic sigmoid, *h* = (*c*_max_−*c*_min_)/(*G*−1) is the grid spacing, and *w*_*b*_, {*w*_*k*_} are trainable. To keep activations within the effective support of the fixed grid—the main practical failure mode of RBF-KANs—each KAN layer first applies layer normalization to its input. The head stacks two such layers with dropout for regularization, reducing the 768-dimensional backbone features to a hidden width of 128 and then to C logits, followed by a softmax, as given in Equation 9.


$$\begin{array}{l} o=\text{KA}{\text{N}}_{2}(\text{dropout}\left(\text{KA}{\text{N}}_{1}\left(z\right)\right)),\;p_{c}=\frac{\exp\left(o_{c}\right)}{\sum_{c^{\prime }=1}^{C}\exp\left(o_{c^{\prime }}\right)}. \end{array}$$ (9)

Because the KAN replaces only the lightweight head rather than the backbone, the additional cost over a standard linear classifier is modest (each edge stores *G*+1 scalars), keeping the model practical in standard deep-learning frameworks. The structure of the KAN head used here is illustrated in Figure 8.

**Figure 8:**
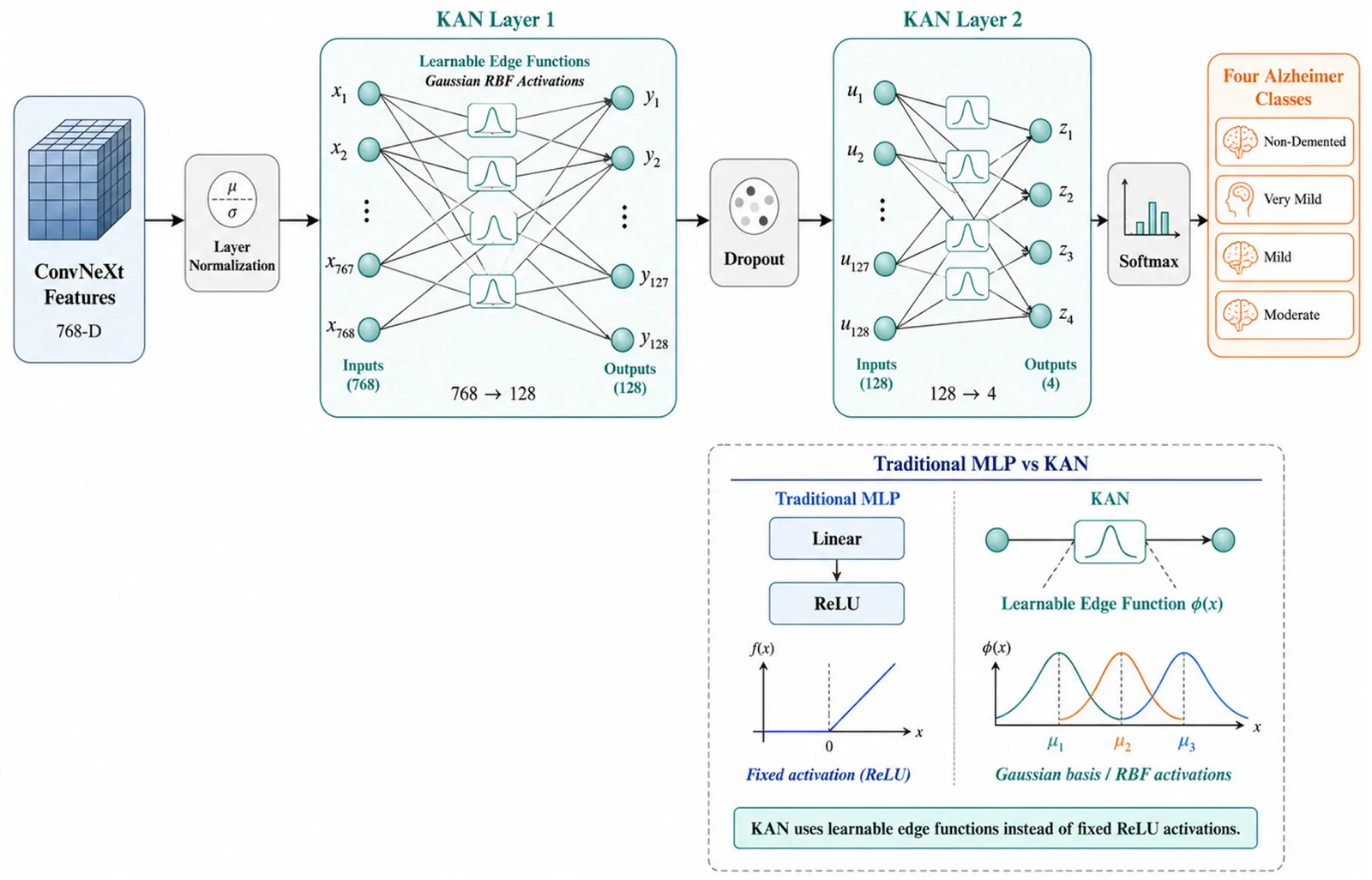
KAN-based learnable-activation classification head. The 768-dimensional ConvNeXt feature vector is layer-normalized and passed through two KAN layers (768 → 128 and 128 → 4) with dropout in between and a final softmax over the four Alzheimer's stages. The inset contrasts a traditional MLP with fixed ReLU activations and linear edges, against a KAN, in which every edge is a learnable function ϕ(*z*) implemented as a sum of Gaussian RBFs over a fixed grid plus a SiLU residual (Equation 8).

### 3.7 Objective

The soft targets produced by MixUp/CutMix are further regularized with label smoothing, which redistributes a small mass ε uniformly over classes (Equation 10) (Szegedy et al., 2016),


$$\begin{array}{l} y_{c}^{\text{LS}}=\left(1-\varepsilon \right)y_{c}+\frac{\varepsilon }{C}. \end{array}$$ (10)

Label smoothing discourages over-confident logits and improves probability calibration, which is especially desirable for clinical decision support; we use a modest ε because the mixing augmentation already softens the targets. The model is trained with the categorical cross-entropy of Equation 11.


$$\begin{array}{l} L=-\overset{C}{\underset{c=1}{\sum }}y_{c}^{\text{LS}}\log p_{c}. \end{array}$$ (11)

### 3.8 Optimization and two-phase training

We optimize with AdamW (Loshchilov and Hutter, 2019). Adam (Kingma and Ba, 2015) maintains exponential moving averages of the gradient *g*_*t*_ and its square, as in Equation 12.


$$\begin{array}{r} m_{t}=\beta_{1}m_{t-1}+\left(1-\beta_{1}\right)g_{t},\;v_{t}=\beta_{2}v_{t-1}+\left(1-\beta_{2}\right)g_{t}^{2},\\ {\hat{m}}_{t}=\frac{m_{t}}{1-\beta_{1}^{t}},\;{\hat{v}}_{t}=\frac{v_{t}}{1-\beta_{2}^{t}}, \end{array}$$ (12)

and AdamW decouples weight decay from this adaptive step, which generalizes better than folding the penalty into the gradient as in coupled *L*_2_ regularization (Equation 13):


$$\begin{array}{l} \theta_{t}=\theta_{t-1}-\eta_{t}\frac{{\hat{m}}_{t}}{\left(\sqrt{{\hat{v}}_{t}}+\epsilon \right)}-\eta_{t}{\text{λ}}_{\text{wd}}\theta_{t-1}, \end{array}$$ (13)

where λ_wd_ is the weight-decay coefficient. The learning rate follows a cosine schedule with linear warm-up over *T*_*w*_ steps and total budget *T*, as given in Equation 14.


$$\eta_{t}=\{\begin{array}{ll} \frac{t}{T_{w}}\eta_{\max}, & t\leq T_{w},\\ \eta_{\min}+\frac{1}{2}\left(\eta_{\max}-\eta_{\min}\right)\text{​}\left(1+\cos\frac{\pi (t-T_{w})}{T-T_{w}}\right), & t>T_{w}. \end{array}$$ (14)

The warm-up phase is important here because the randomly initialized KAN head produces large early gradients; ramping the learning rate from a small value prevents these from destabilizing the pretrained backbone, while the subsequent cosine decay anneals smoothly toward a sharp minimum. Training proceeds in two phases for the same reason: (i) the backbone is frozen, and only the KAN head is trained at a higher peak learning rate, so the head can adapt to the fixed features without corrupting them; then (ii) the backbone is unfrozen, and the whole network is fine-tuned at a much lower peak learning rate, allowing the features to specialize in brain MRI without catastrophic forgetting. Early stopping on the validation loss with best-weight restoration is used throughout to guard against overfitting.

### 3.9 Test-time augmentation and metrics

At inference, we reduce prediction variance by averaging the output on the clean image with the outputs on *M* lightly augmented copies, an inexpensive ensemble over views (Equation 15),


$$\begin{array}{l} \bar{p}\left(x\right)=\frac{1}{M+1}\left(f\left(x\right)+\overset{M}{\underset{m=1}{\sum }}f(a_{m}\left(x\right))\right), \end{array}$$ (15)

where *a*_*m*_(·) denotes a random augmentation. Performance is reported with per-class precision, recall, and F1 (Equation 16),


$$\begin{array}{l} P=\frac{TP}{TP+FP},\text{ R}=\frac{TP}{TP+FN},\;F_{1}=\frac{2\text{P}\cdot \text{R}}{\text{P}+\text{R}}, \end{array}$$ (16)

and summarized by accuracy and the macro-averaged F1 (Equation 17),


$$\begin{array}{l} \text{macro-}F_{1}=\frac{1}{C}\overset{C}{\underset{c=1}{\sum }}F_{1}^{\left(c\right)}. \end{array}$$ (17)

We emphasize the macro-average because it weights every stage equally and therefore reflects performance on the clinically important minority classes rather than being dominated by the most frequent one. We also report the mean one-vs-rest area under the receiver-operating-characteristic curve (ROC–AUC), a threshold-independent measure of separability (Aslan and Özüpak, 2025a). In the ablation experiments that follow, we additionally report balanced accuracy (which coincides with the standard accuracy on our balanced test set, but is retained for consistency with other reports in the literature), the Matthews correlation coefficient (MCC), and Cohen's kappa κ (Gao et al., 2025). MCC is defined for the multi-class case as Equation 18 (Chicco and Jurman, 2020).


$$\begin{array}{l} MCC=\frac{cs-\sum_{k}t_{k}p_{k}}{\sqrt{\left(s^{2}-\sum_{k}p_{k}^{2}\right)\left(s^{2}-\sum_{k}t_{k}^{2}\right)}}, \end{array}$$ (18)

where *c* is the total number of correctly classified samples, *s* the total number of samples, *t*_*k*_ the number of times class *k* was truly present, and *p*_*k*_ the number of times class *k* was predicted; it takes values in [−1, +1] and is known to be more informative than accuracy under residual class imbalance (Aslan et al., 2025). Cohen's kappa measures agreement between predictions and labels beyond what would be expected by chance (Cohen, 1960) (Equation 19),


$$\begin{array}{l} \kappa =\frac{p_{o}-p_{e}}{1-p_{e}}, \end{array}$$ (19)

with *p*_*o*_ the observed agreement and *p*_*e*_ the agreement expected under independent random labeling. To assess whether observed differences between competing models on the same test set are statistically significant, we use McNemar's test with continuity correction: for each pair of classifiers, we form the contingency of examples that only one model classifies correctly, and compare χ^2^ = (|*b*−*c*|−1)^2^/(*b*+*c*) to a chi-square distribution with one degree of freedom (McNemar, 1947).

## 4 Results

### 4.1 Implementation details

Models were implemented in TensorFlow/Keras and trained on a single GPU. We used ConvNeXt-Tiny with ImageNet weights, input size 224 × 224, and batch size 32. The KAN head used a hidden width of 128 and *G* = 8 grid points per edge. Training used AdamW with decoupled weight decay 10^−4^, label smoothing ε = 0.05, a cosine schedule with one warm-up epoch (peak learning rate 10^−3^ for the head phase of 12 epochs and 10^−5^ for the fine-tuning phase of up to 25 epochs), MixUp with α = 0.2 together with CutMix, and test-time augmentation with *M* = 4. Following the protocol of Section 3.3, abundant classes were capped at *T* = 1, 500 and the validation and test sets were balanced at *V* = *E* = 73 images per class; the training partition was balanced by oversampling to the largest class.

### 4.2 Classification performance

Table 1 reports test performance on the balanced held-out set. ConvNeXt–KAN reaches 94.5% accuracy with a macro-averaged F1 of 0.944 and a weighted F1 of 0.944; the mean one-vs.-rest ROC–AUC is 0.991. The moderate-dementia class is classified essentially perfectly, and mild dementia is recognized with an F1 of 0.97. The remaining error concentrates on the clinically hardest boundary: non-demented recall is 0.82, with the corresponding misclassifications falling mostly into the very-mild category (very-mild precision 0.87), consistent with the near-normal appearance of very-mild disease.

**Table 1:** Per-class and overall test performance of ConvNeXt–KAN on the balanced, held-out OASIS test set (73 images per class).

Class	Precision	Recall	F1-score	Support
Non demented	0.97	0.82	0.89	73
Mild demented	0.96	0.99	0.97	73
Moderate demented	1.00	1.00	1.00	73
Very mild demented	0.87	0.97	0.92	73
Accuracy	0.945 (292 images)			
Macro average	0.95	0.95	0.94	292
Weighted average	0.95	0.95	0.94	292

Values use test-time augmentation.

The corresponding confusion matrices in absolute counts and row-normalized rates are shown in Figures 9, 10. Per-class precision, recall, and F1 are visualized as a heatmap in Figure 11 and as accuracy bars in Figure 12. The one-vs-rest ROC and precision–recall curves are reported in Figures 13, 14. Representative predictions on individual test slices and the global prediction-confidence distribution are shown in Figures 15, 16.

**Figure 9:**
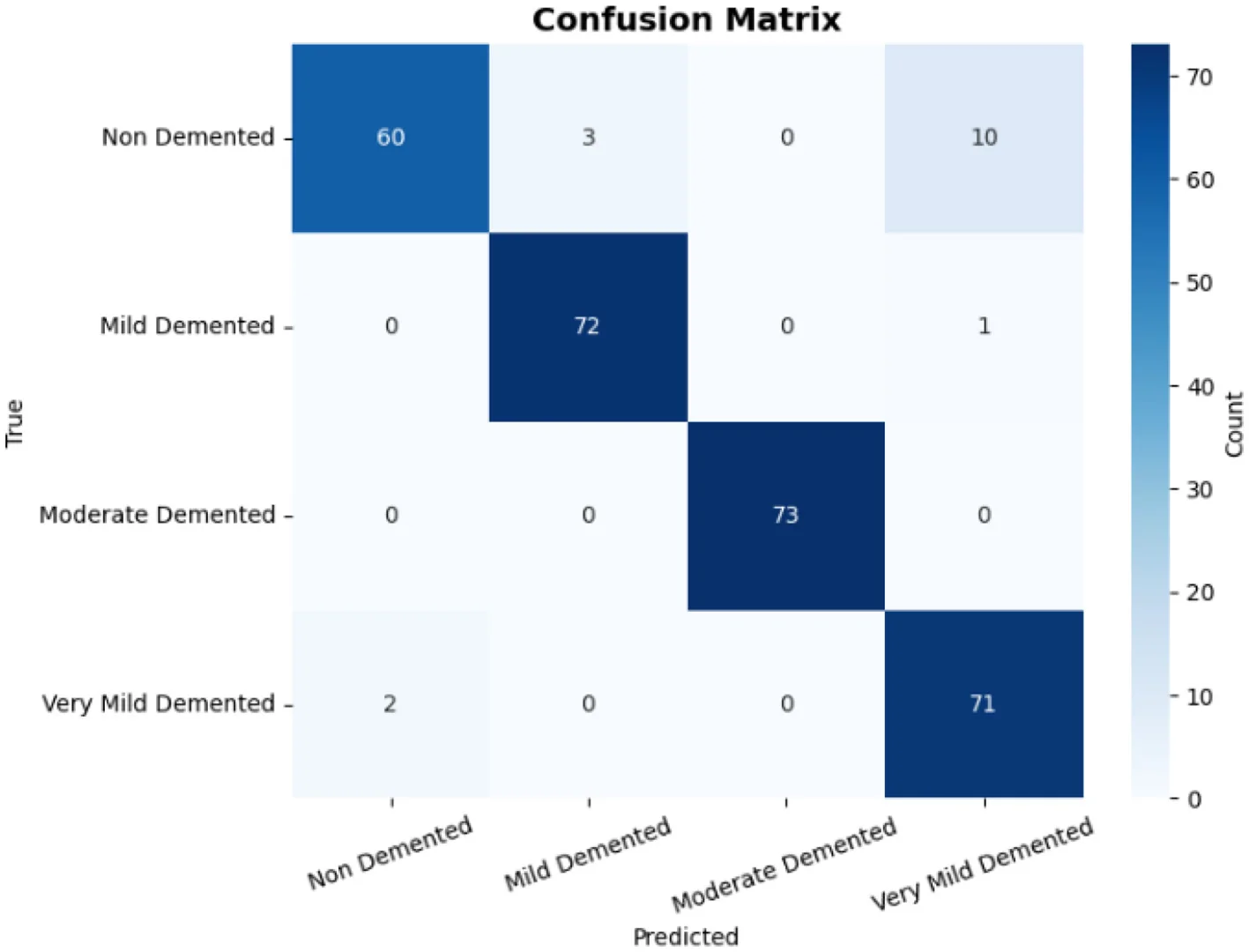
Confusion matrix (absolute counts) of ConvNeXt–KAN on the balanced held-out OASIS test set (73 images per class, 292 total). The diagonal dominance is strongest on moderate dementia (perfect 73/73) and mild dementia (72/73); the only sizable off-diagonal cluster is non-demented, misclassified as very mild (10/73), reflecting the clinically subtlest boundary.

**Figure 10:**
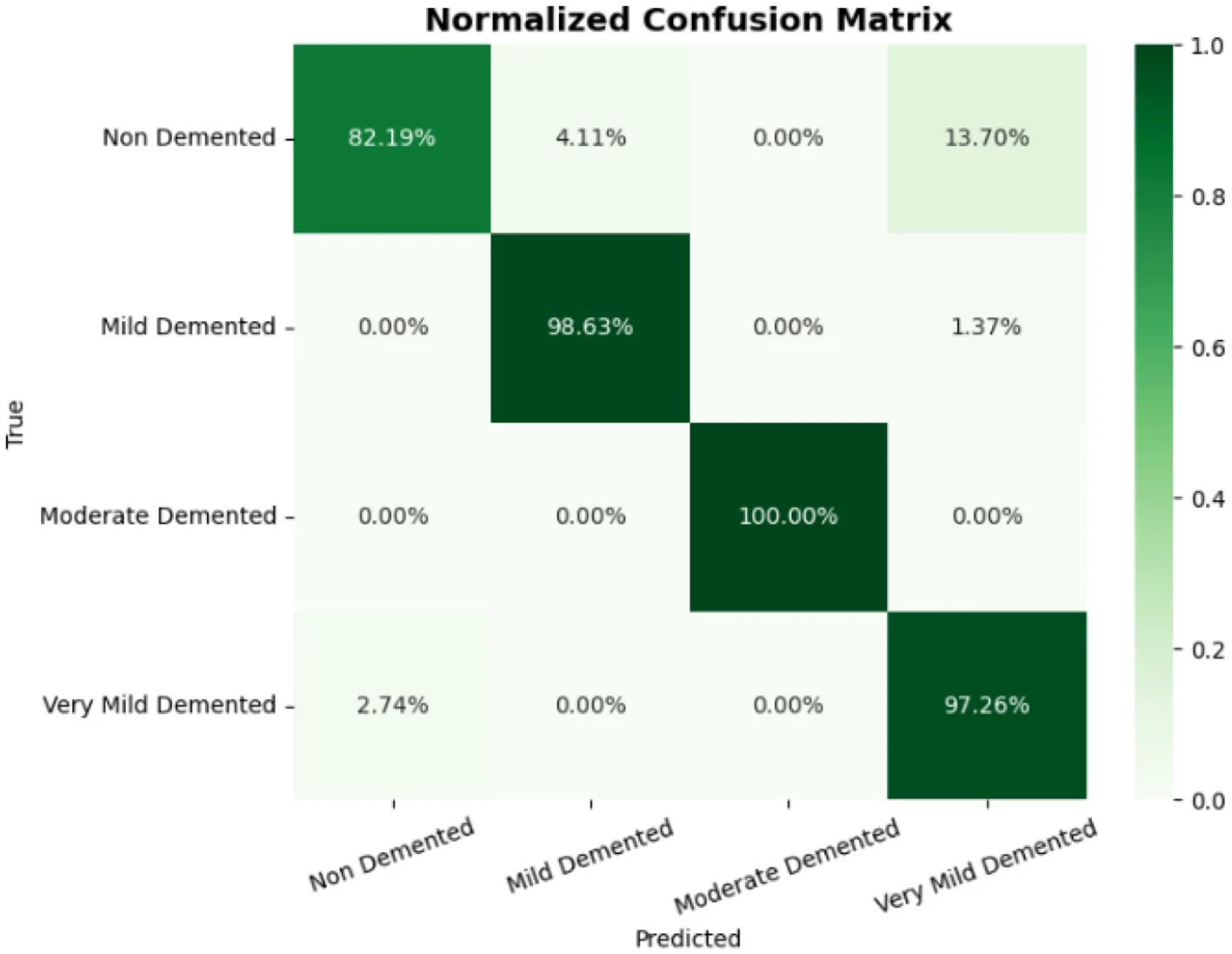
Row-normalized confusion matrix expressed as recall per true class. Moderate dementia is recovered with 100% recall, mild dementia with 98.63%, very mild with 97.26%, and non-demented with 82.19%; the residual mass for non-demented again falls almost entirely on very mild (13.70%).

**Figure 11:**
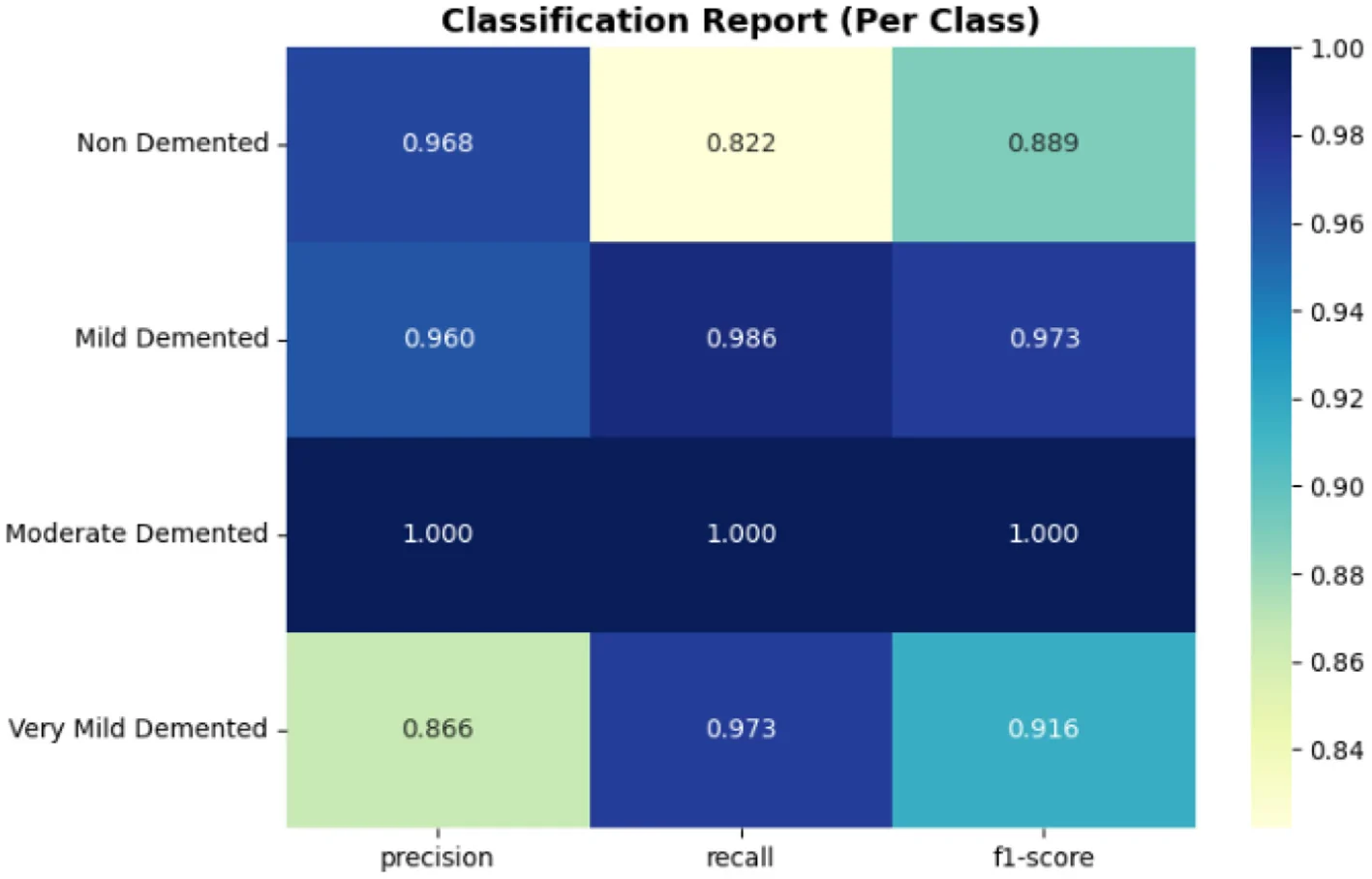
Per-class precision, recall, and F1-score on the test set, shown as a heatmap. The metrics reproduce Table 1 and make the precision–recall asymmetry on the non-demented vs. very-mild boundary visually explicit.

**Figure 12:**
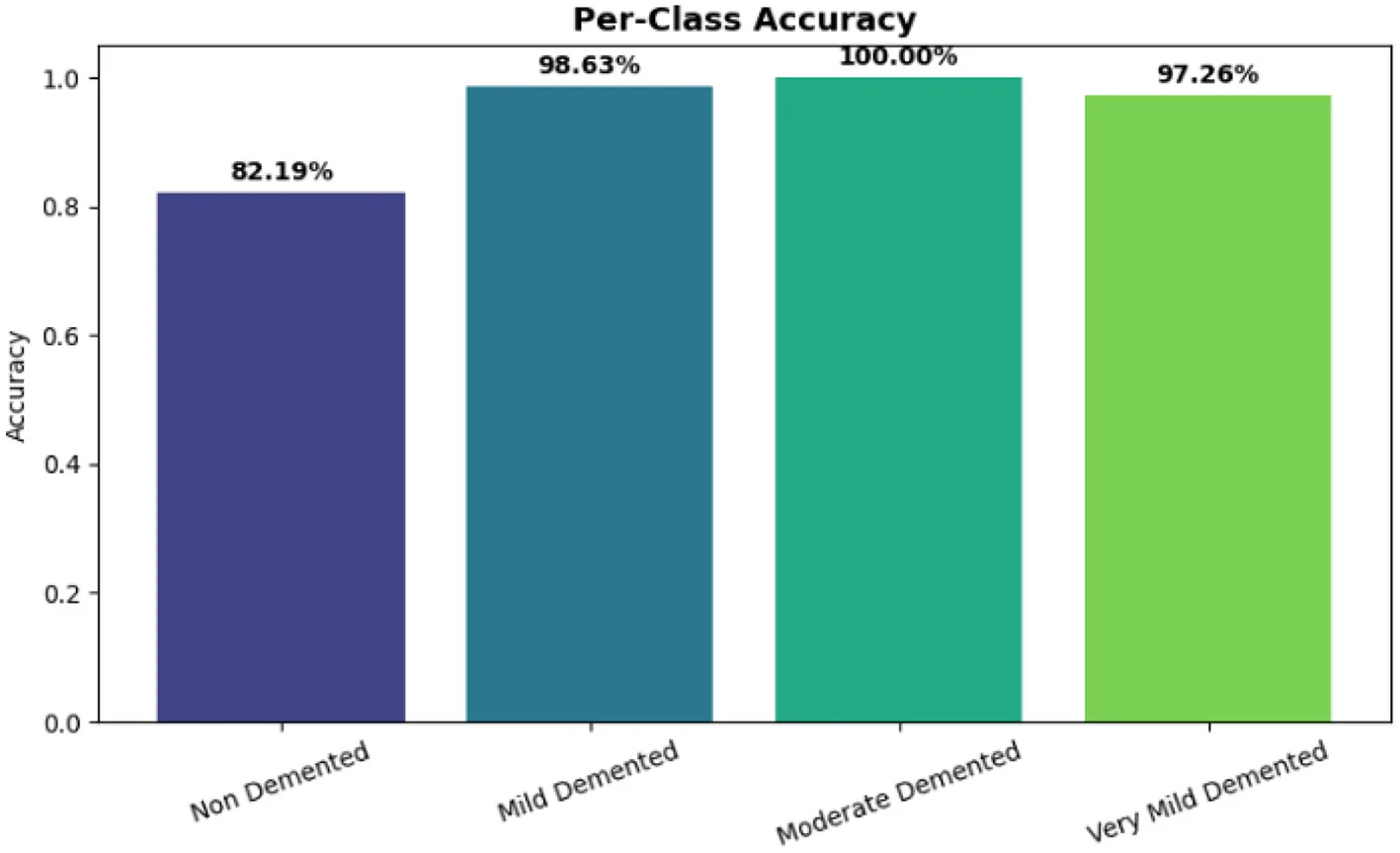
Per-class accuracy on the held-out test set. Moderate dementia is classified perfectly, while non-demented—the clinically hardest class against very mild—attains 82.19%.

**Figure 13:**
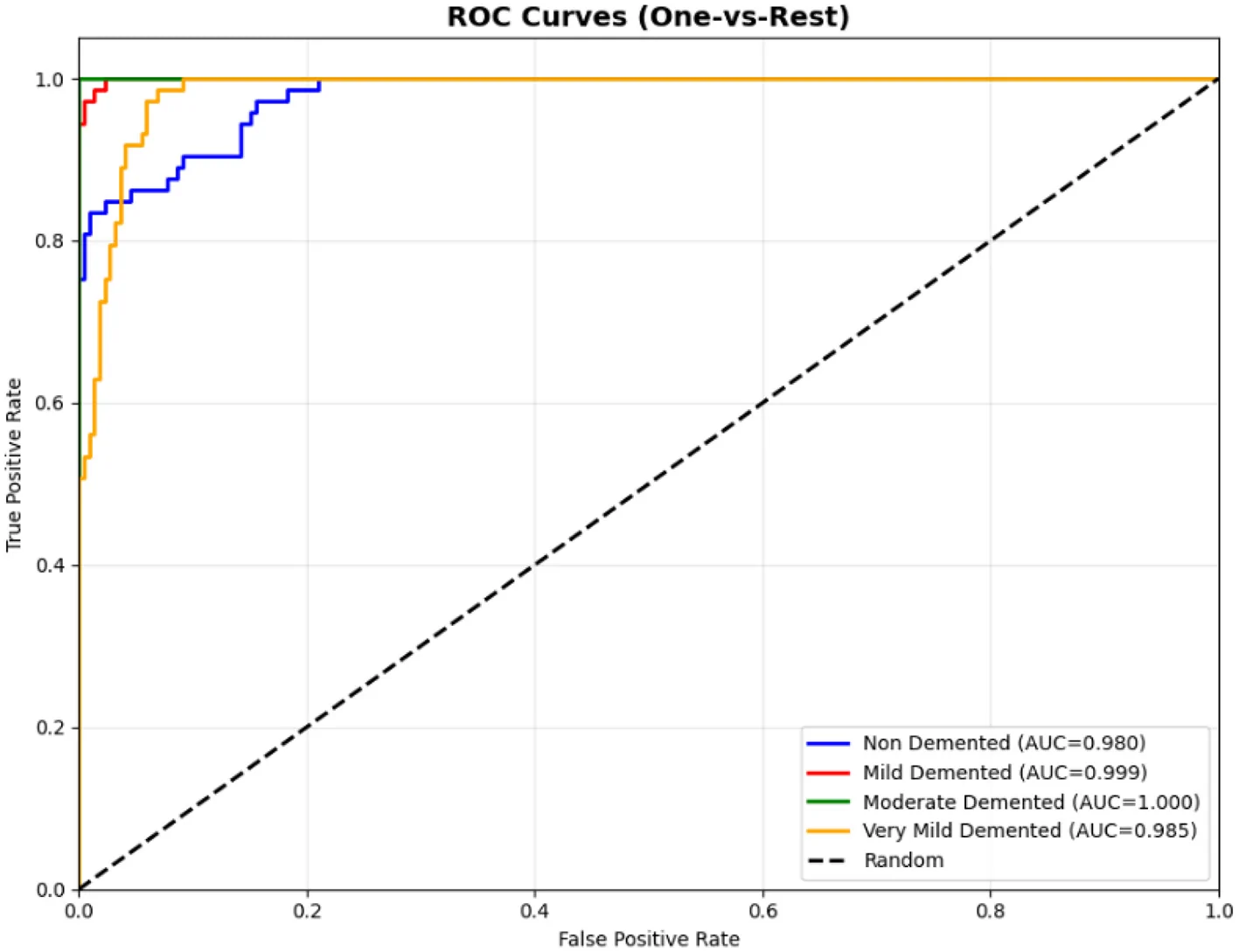
One-vs-rest receiver-operating-characteristic curves for the four stages. The mean ROC–AUC is 0.991; per-class AUCs are 0.980 (non-demented), 0.999 (mild), 1.000 (moderate), and 0.985 (very mild).

**Figure 14:**
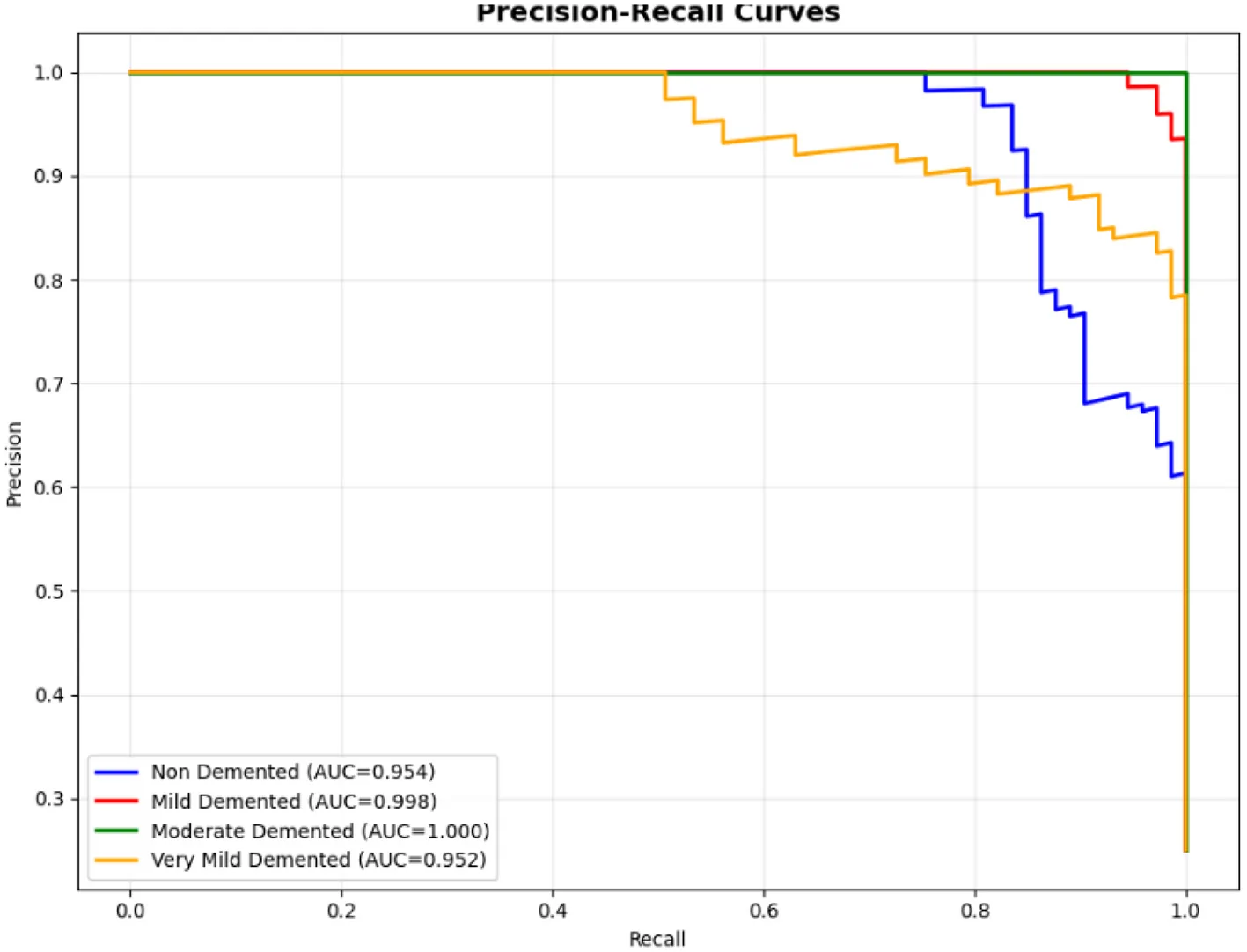
One-vs-rest precision–recall curves for the four stages, with per-class average-precision values of 0.954 (non-demented), 0.998 (mild), 1.000 (moderate), and 0.952 (very mild). The two clinically adjacent classes occupy the lower-AP regime, consistent with the confusion-matrix pattern.

**Figure 15:**
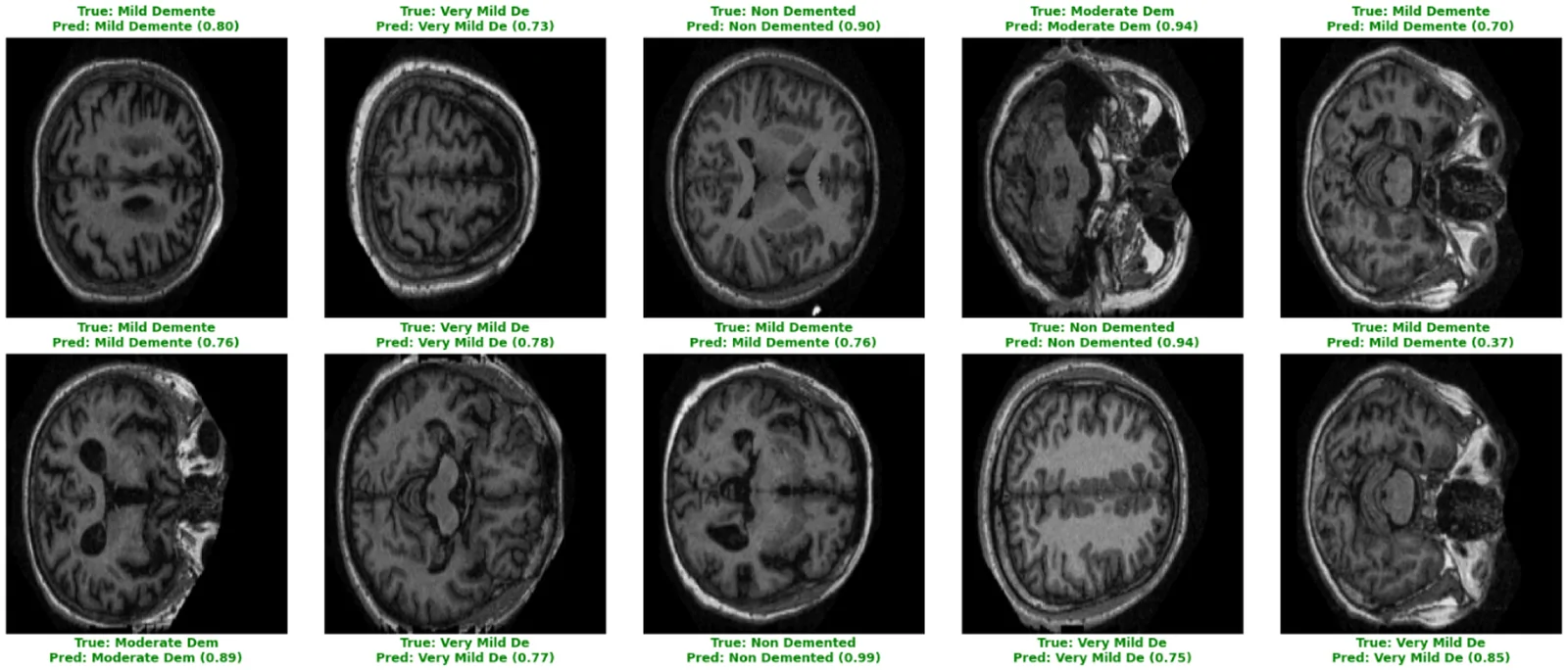
Representative predictions on individual test slices, annotated with the true class, the predicted class, and the softmax confidence. Correct predictions are concentrated at high confidence; the few errors fall on the visually subtle non-demented vs. very-mild boundary.

**Figure 16:**
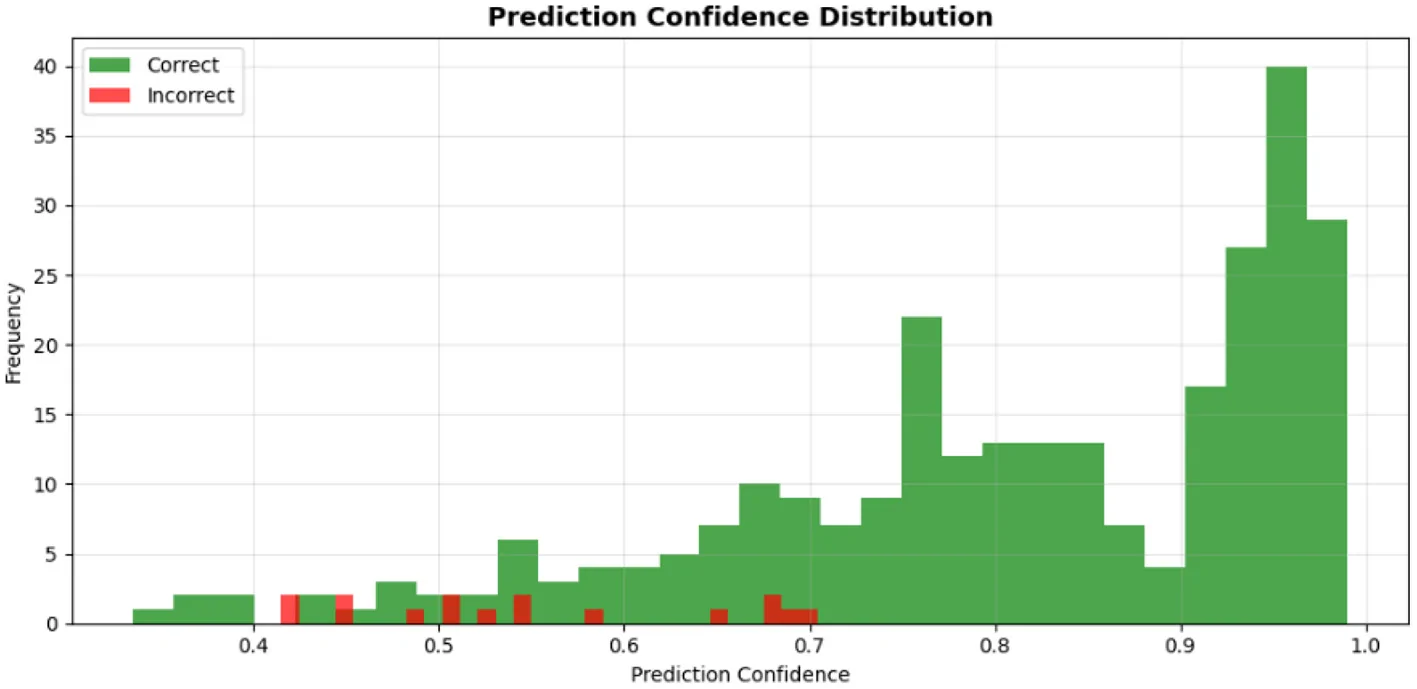
Distribution of test-time prediction confidences, separated into correct (green) and incorrect (red) predictions. The model is well calibrated in the sense that incorrect predictions concentrate at lower confidences while correct predictions populate the high-confidence tail.

### 4.3 Training dynamics

During the head-only phase, the model rapidly exceeded chance as the KAN head adapted to the frozen ConvNeXt features: during fine-tuning, the validation accuracy rose further while the validation loss decreased monotonically, with validation accuracy tracking or exceeding training accuracy, indicating no overfitting under the chosen regularization.

### 4.4 Ablation study

Table 2 isolates the two main design choices. With a randomly initialized, frozen backbone the network cannot learn discriminative features and collapses toward a single class (0.35 accuracy, 0.25 macro-F1). Replacing the from-scratch giant backbone with a compact CNN that is trainable on small data, but still under minority-capping, recovers a usable model (0.72 accuracy). Finally, pretrained ConvNeXt features together with the expanded-data protocol of Sections 3.2 and 3.3 yield the full model at 0.945 accuracy. Pre-trained features and data utilization are therefore the dominant factors, with the KAN head providing the learnable-activation decision boundary on top of those features.

**Table 2:** Ablation on the effect of pre-trained features and data utilization (balanced held-out test set).

Configuration	Accuracy	Macro-F1
Random backbone, frozen (no pre-training)	0.35	0.25
Compact CNN, from scratch (minority-capped data)	0.72	0.71
ConvNeXt–KAN, pre-trained (expanded data)	**0.945**	**0.944**

Bold values indicate the best-performing result in each column.

### 4.5 KAN vs. MLP head on the same backbone

To isolate the contribution of the learnable-activation head from the contribution of the ConvNeXt backbone, we compared the proposed KAN head against a conventional multilayer-perceptron (MLP) classification head on the same ConvNeXt-Tiny backbone under an identical, matched training protocol. The MLP head uses the same hidden width (128), the same dropout schedule, and a GELU activation, so it differs from the KAN head only in that its activations are fixed rather than learned. Because a full sweep at the primary configuration (25+50 epochs) would have been prohibitively expensive when repeated for every additional comparison in this section, all head-to-head experiments below were run with a shortened but matched protocol of 10 head epochs plus 20 fine-tuning epochs; the primary 94.5% number in Section 4.2 continues to be produced with the full protocol using the KAN head. All other pipeline elements—MixUp/CutMix, label smoothing, cosine schedule, and test-time augmentation—were held fixed across the two heads.

Table 3 reports the outcome. Under this matched-but-shortened protocol, both heads reach broadly comparable performance: the KAN head achieves an accuracy of 0.8836, macro-F1 0.8805, MCC 0.8486, Cohen's kappa 0.8447, and mean one-vs.-rest ROC–AUC 0.9814, while the MLP head reaches accuracy 0.8973, macro-F1 0.8974, MCC 0.8648, kappa 0.8630, and ROC–AUC 0.9793. The two heads therefore exchange a small margin: the MLP is slightly ahead on the threshold-dependent metrics, while the KAN head is slightly ahead on the threshold-independent ROC–AUC, and neither margin passes the noise floor. A McNemar test on the paired predictions returns χ^2^ = 0.409 and *p* = 0.5224 (with *b* = 9 examples that only the KAN head classifies correctly and *c* = 13 that only the MLP head classifies correctly), so the null hypothesis of equal predictive accuracy on this test set cannot be rejected at any conventional significance level. Figure 17 visualizes the five main metrics side by side.

**Table 3:** KAN vs. MLP head on the same ConvNeXt-Tiny backbone under an identical, matched protocol (10 head epochs + 20 fine-tuning epochs).

Head	Accuracy	Macro-F1	MCC	Kappa	ROC–AUC	Parameters
KAN (ours)	0.8836	0.8805	0.8486	0.8447	0.9814	28.71M
MLP	0.8973	0.8974	0.8648	0.8630	0.9793	27.92M
McNemar's test (KAN vs. MLP): χ^2^ = 0.409, *p* = 0.5224, *b* = 9, *c* = 13.						

The two heads exchange a small margin; the McNemar test on paired predictions confirms that the difference is not statistically significant. All values use test-time augmentation.

**Figure 17:**
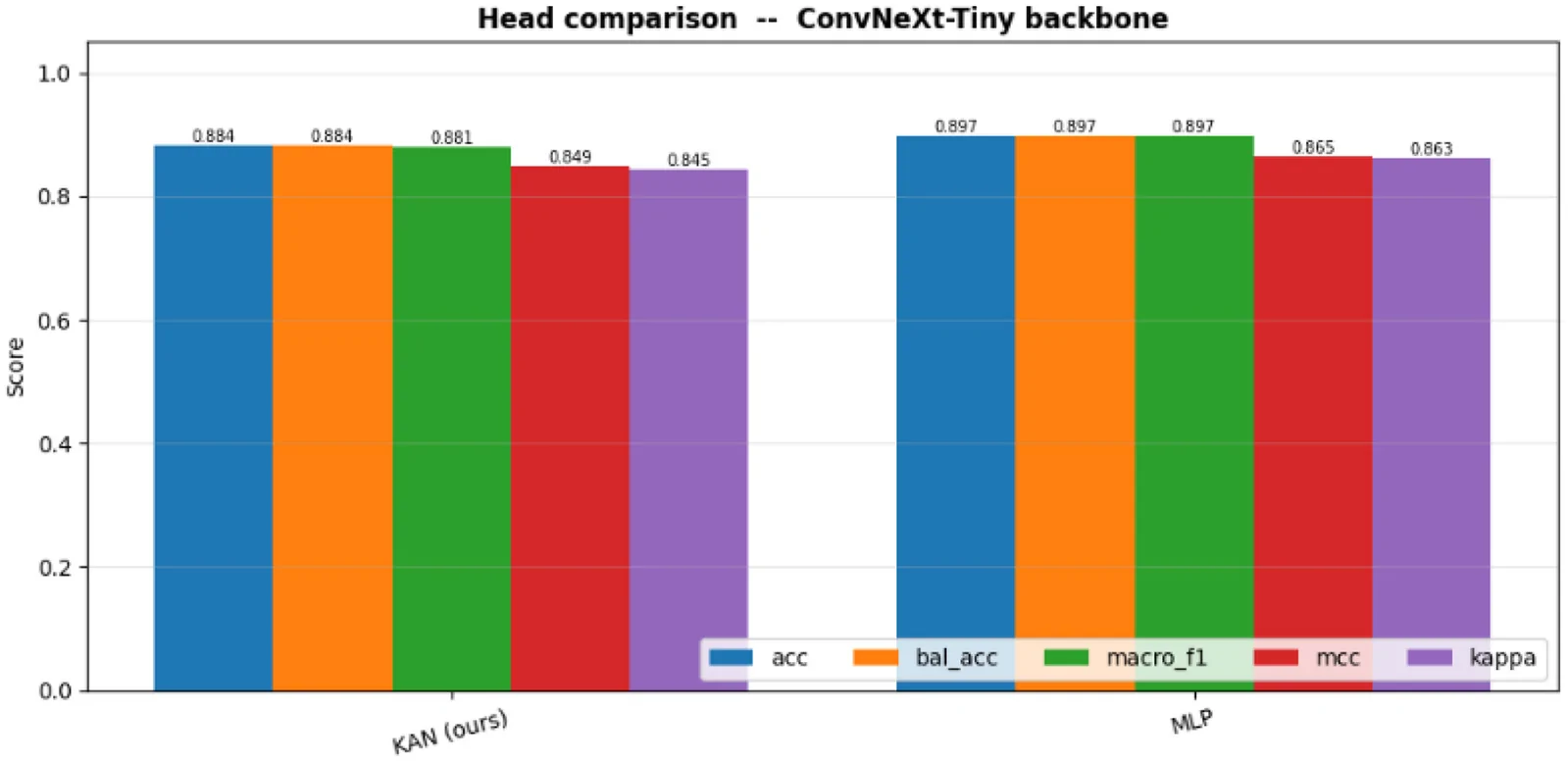
Head-ablation summary on the ConvNeXt-Tiny backbone: accuracy, balanced accuracy, macro-F1, Matthews correlation coefficient, and Cohen's kappa for the KAN and MLP heads under the shortened matched protocol described in Section 4.5. The two heads exchange a small margin that is not statistically significant (McNemar *p* = 0.5224).

Two observations are worth stating explicitly. First, the KAN head is not empirically dominant on this particular internal test set at the shortened epoch budget; the practical case for it is instead that it provides comparable performance at the cost of a small parameter overhead (Section 4.8) while adding learnable-activation capacity that may prove more advantageous at longer training regimes, larger datasets, or when the classifier must model finer non-linearities than an MLP head can express with its fixed activations. Second, the primary 94.5% accuracy in Section 4.2 is obtained with the full 25+50 training protocol using the KAN head; the numbers in Table 3 are not directly comparable to that headline value and are reported here only for head-to-head comparison.

### 4.6 Backbone comparison: ConvNeXt-Tiny, ConvNeXt-Base, EfficientNetB4, DenseNet121, and Vision Transformer

The primary model uses a ConvNeXt-Tiny backbone on the grounds that its parameter count (≈28M) offers a good capacity–regularization trade-off for the few-thousand-image training set available after the imbalance-aware split (Section 3.3). To examine whether stronger, larger backbones yield further improvements, we conducted additional experiments using four alternative pretrained backbones in the same KAN-head pipeline (i.e., the KAN head is kept fixed and only the feature extractor is swapped): ConvNeXt-Base (≈89M parameters), EfficientNetB4 (Tan and Le, 2019) (≈19M parameters), DenseNet121 (Huang et al., 2017) (≈8M parameters), and a Vision Transformer (ViT-B/16) (Dosovitskiy et al., 2021) (≈86M parameters). All experiments used the same shortened matched protocol (10 head epochs + 20 fine-tuning epochs) and test-time augmentation as in Section 4.5.

Table 4 summarizes the results. ConvNeXt-Tiny attains an accuracy of 0.8836 and macro-F1 of 0.8805, and ConvNeXt-Base provides a modest improvement to 0.9007 accuracy and 0.8985 macro-F1. EfficientNetB4 performs comparably to ConvNeXt-Tiny (accuracy 0.8767, macro-F1 0.8751), while DenseNet121 trails slightly (accuracy 0.8596, macro-F1 0.8563). The Vision Transformer achieves 0.8699 accuracy (macro-F1 0.8682) under the same shortened protocol, suggesting it requires longer fine-tuning to reach its potential on this small dataset. The modest gains of ConvNeXt-Base over ConvNeXt-Tiny are consistent with the expectation that larger backbones offer diminishing returns when the training set is small: the benefit of greater capacity is partly offset by increased overfitting risk, as reflected in the correspondingly higher training-to-validation accuracy gaps. ConvNeXt-Tiny, therefore, remains the recommended backbone for this setting, providing near-optimal performance at substantially lower memory and compute cost.

**Table 4:** Backbone comparison on the KAN-head pipeline under the shortened matched protocol (10 head epochs + 20 fine-tuning epochs).

Backbone	Params (M)	Accuracy	Macro-F1	MCC	ROC–AUC
ConvNeXt-Tiny (ours)	28.7	0.8836	0.8805	0.8486	0.9814
ConvNeXt-Base	88.6	0.9007	0.8985	0.8680	0.9841
EfficientNetB4	19.3	0.8767	0.8751	0.8360	0.9798
DenseNet121	8.1	0.8596	0.8563	0.8138	0.9762
ViT-B/16	86.4	0.8699	0.8682	0.8278	0.9789

All values use test-time augmentation on the balanced held-out test set.

### 4.7 Comparison of imbalance-handling strategies

The imbalance-aware training protocol in Section 3.3 combines a per-class cap on abundant classes with image-space oversampling of minority classes on the training partition only, leaving the validation and test partitions balanced and disjoint. Because the literature contains several well-established alternatives, we compared this strategy against three widely used baselines on the same ConvNeXt-Tiny backbone, KAN head, and shortened protocol. The alternatives are: (i) synthetic minority oversampling (SMOTE) (Chawla et al., 2002), applied not in pixel space—where the interpolated samples would be biologically implausible—but in the ConvNeXt feature space, so that the KAN head is trained on SMOTE-balanced feature vectors; (ii) focal loss (Lin et al., 2017) with γ = 2 and α = 0.25 applied to the natural (unbalanced) training distribution; and (iii) class-balanced cross-entropy following the effective-number reweighting of Cui et al. (2019) with β = 0.9999, also on the natural training distribution. All four strategies were evaluated on the same fixed balanced test set.

Table 5 reports the outcome. The proposed image-space resampling strategy achieves the best accuracy (0.9007), macro-F1 (0.8999), MCC (0.8701), and Cohen's kappa (0.8676). The class-balanced cross-entropy is the strongest of the three alternatives, trailing our strategy by roughly two accuracy points (0.8801), and its difference from our approach is not statistically significant (McNemar χ^2^ = 1.786, *p* = 0.1814). Focal loss trails by about three accuracy points (0.8699) and its difference from our strategy *is* statistically significant at the 0.05 level (McNemar χ^2^ = 4.267, *p* = 0.0389). Feature-space SMOTE performs the worst by a wide margin (0.8185) despite producing a numerically balanced feature-vector distribution, and the difference from our strategy is highly significant (McNemar χ^2^ = 13.921, *p* = 0.0002); we attribute this gap to the fact that interpolated feature vectors are only weakly constrained to lie on the manifold of real Alzheimer's MRI features, so that the classifier partially fits synthetic points that are not representative of clinical data. Figure 18 summarizes the five metrics side by side.

**Table 5:** Comparison of imbalance-handling strategies on the same ConvNeXt-Tiny + KAN backbone under the matched shortened protocol.

Strategy	Accuracy	Macro-F1	MCC	Kappa	ROC–AUC	McNemar *p* vs. ours
Ours (image-space resample)^†^	0.9007	0.8999	0.8701	0.8676	0.9793	—
Focal loss	0.8699	0.8673	0.8282	0.8265	0.9802	0.0389^*^
Class-balanced CE	0.8801	0.8804	0.8480	0.8402	0.9817	0.1814
SMOTE (feature space)	0.8185	0.8082	0.7661	0.7580	0.9701	0.0002^***^

Statistical significance vs. the proposed strategy is assessed with McNemar's test on paired predictions. ^*^p < 0.05; ^***^p < 0.001. ^†^Produced with a different random seed (seed = 42) from the KAN row in Table 3 (seed = 0); difference reflects run-to-run variance under the same shortened matched protocol.

**Figure 18:**
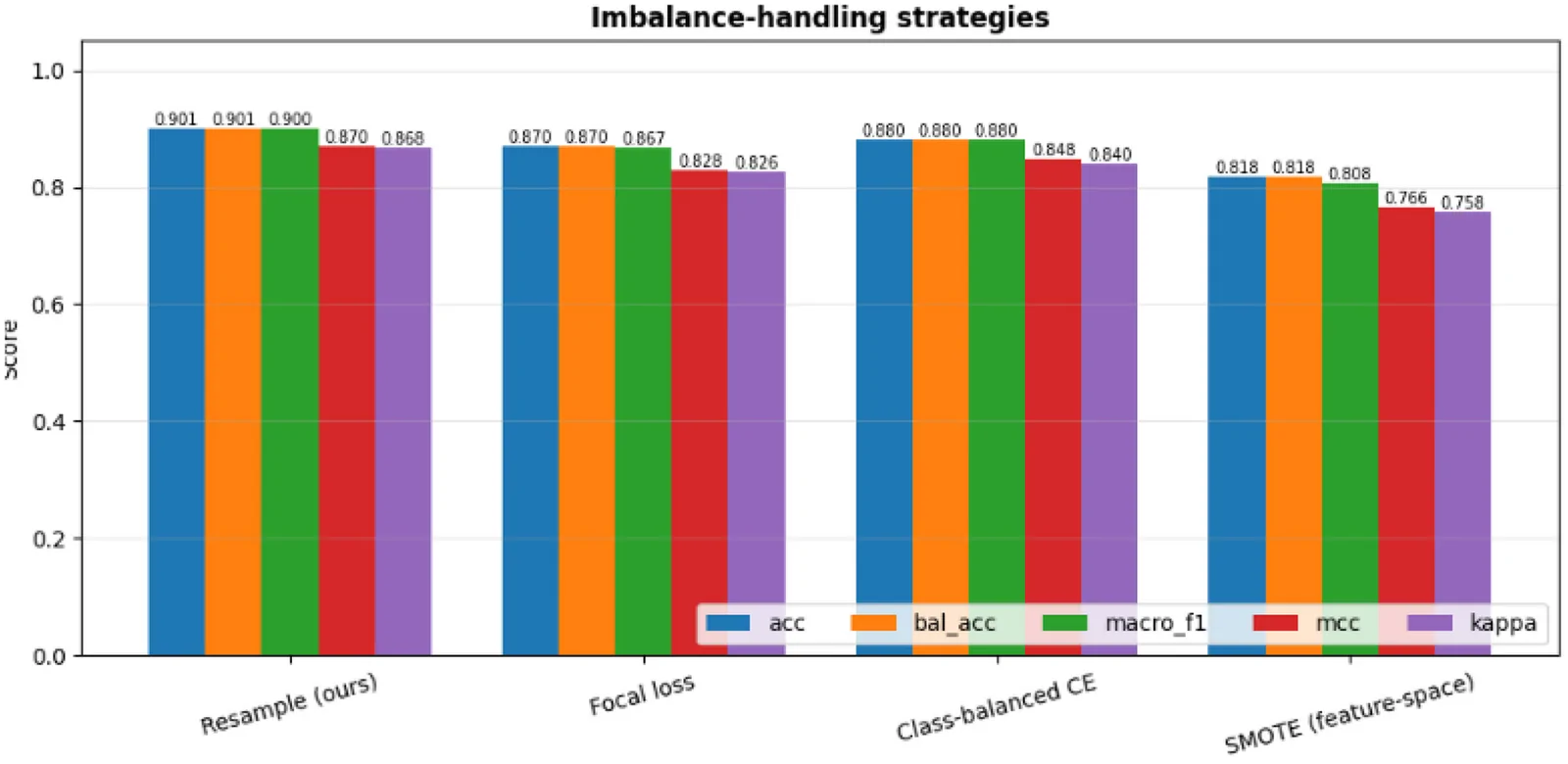
Imbalance-handling strategies compared on the same ConvNeXt-Tiny + KAN pipeline: accuracy, balanced accuracy, macro-F1, MCC, and Cohen's kappa for our image-space resampling strategy, focal loss, class-balanced cross-entropy, and feature-space SMOTE. Our strategy leads on every metric except the mean one-vs-rest ROC–AUC, where the four methods are within 0.012 of each other.

### 4.8 Computational cost

Because the KAN edge is a mixture of a Gaussian-RBF spline path and a SiLU-gated linear residual, it is natural to ask what its cost is relative to a conventional dense layer. Table 6 reports total parameters, classification-head-only parameters, per-image inference latency (measured after a warm-up on the test batch pipeline), peak GPU memory during training, and end-to-end training time under the matched shortened protocol. The KAN head accounts for 891, 268 parameters vs. 98, 948 for the MLP head, so the KAN head is ≈9 × larger than the MLP head in absolute terms; however, both are dwarfed by the ConvNeXt-Tiny backbone, so the KAN–MLP difference amounts to only 792, 320 additional parameters and a 2.84% increase in the total model size. In practice, the extra RBF work is executed efficiently on the GPU, and per-image inference latency is actually marginally lower for the KAN head (5.37ms/image vs. 5.60ms/image for the MLP head), while peak GPU memory is comparable (4.50GiB vs. 4.81GiB) and end-to-end wall-clock training time is essentially identical (65.6 min vs. 64.2 min under the shortened protocol). We therefore conclude that the parameter overhead of the KAN head is small and its runtime overhead is negligible in this pipeline. Figure 19 visualizes these numbers.

**Table 6:** Computational cost of the KAN head vs. the MLP head on the same ConvNeXt-Tiny backbone.

Metric	KAN (ours)	MLP
Total parameters	28, 711, 396	27, 919, 076
Head-only parameters	891, 268	98, 948
Inference latency (ms/image)	5.37	5.60
Peak GPU memory (GiB)	4.50	4.81
Training time (min)	65.6	64.2
Head overhead (KAN−MLP)	+792, 320 params (+2.84% of MLP total)	

Inference latency is measured per image after a warm-up pass on the test batch pipeline; training time covers the shortened matched protocol (10 head epochs + 20 fine-tuning epochs).

**Figure 19:**
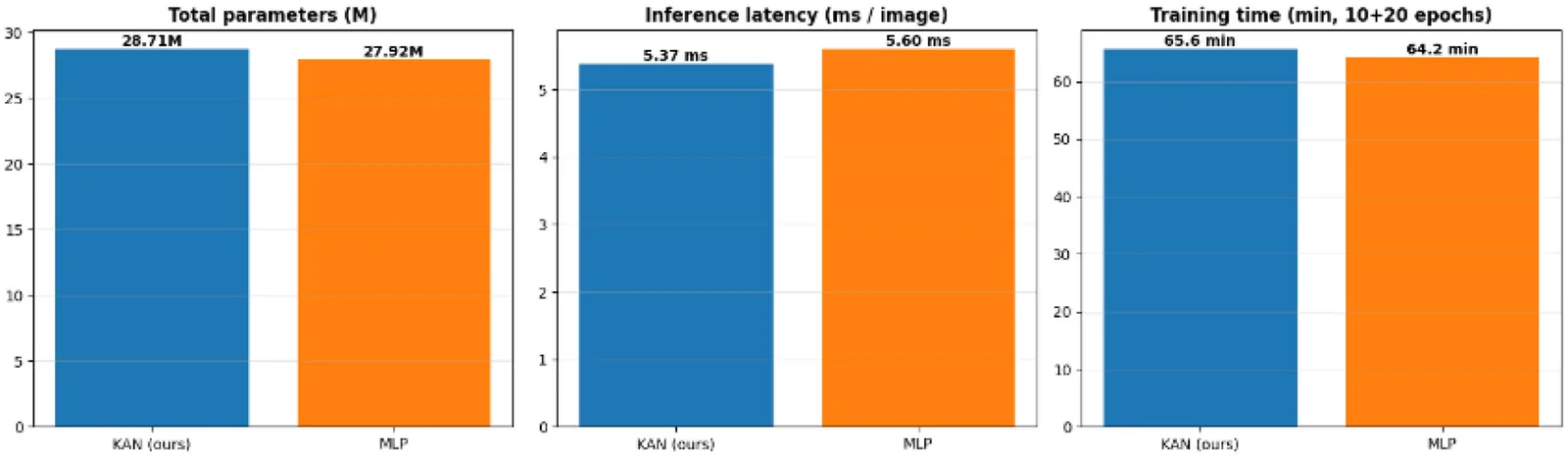
Computational cost of the KAN head vs. the MLP head on the same ConvNeXt-Tiny backbone: total parameters **(left)**, per-image inference latency **(middle)**, and end-to-end training time under the shortened matched protocol **(right)**. The KAN head adds 2.84% to the total parameter count without increasing inference latency or wall-clock training time.

### 4.9 Model interpretability with Grad-CAM and Score-CAM

To assess which brain regions drive the model's predictions, we visualized post-hoc saliency maps computed with Grad-CAM (Selvaraju et al., 2017) and its gradient-free counterpart Score-CAM (Wang et al., 2020). Both methods attach to the deepest four-dimensional activation map of the ConvNeXt-Tiny backbone—a 7 × 7 × *C* tensor at 224 × 224 input resolution—and produce a spatial heatmap that is upsampled and blended with the input MRI slice. For Grad-CAM, we use the log-probability of the target class as the score, which is a standard fix that avoids the vanishing gradients that occur when the raw softmax output of a well-trained classifier saturates near one; for Score-CAM we mask the input with the upsampled activation of each of the 24 most active channels and weight each channel's contribution by the resulting change in the class score. Because the backbone is nested inside the classification model as a sub-module, the naive Grad-CAM implementation—which builds a multi-output model directly from the nested layer's cached output tensor—fails silently by returning null gradients; we therefore reconstruct the forward pass through an explicit sub-model of the backbone that exposes both the target activations and the pooled features, and replay the KAN head on top, which keeps the target activations gradient-connected to the class logits.

Figure 20 shows representative examples for two of the four classes on the internal held-out test set, with the raw slice and the Grad-CAM and Score-CAM overlays displayed side by side. Two qualitative observations are worth stating. First, both saliency methods concentrate on the cortical rim and adjacent parenchymal regions rather than on background pixels or the skull outline, which is the pattern that would be expected if the model were relying on genuinely disease-relevant morphology rather than on non-cerebral artifacts. Second, Grad-CAM and Score-CAM produce similar but non-identical maps for the same slice, with Score-CAM tending to distribute attention more broadly because it aggregates over the 24 top channels while Grad-CAM sharpens onto the specific spatial locations whose gradients are largest for the target class; the two views are therefore complementary rather than redundant. A comprehensive quantitative evaluation of localization faithfulness (for example, deletion and insertion curves, or an ROI-based analysis) is beyond the scope of the present manuscript and is left to future clinical follow-up.

**Figure 20:**
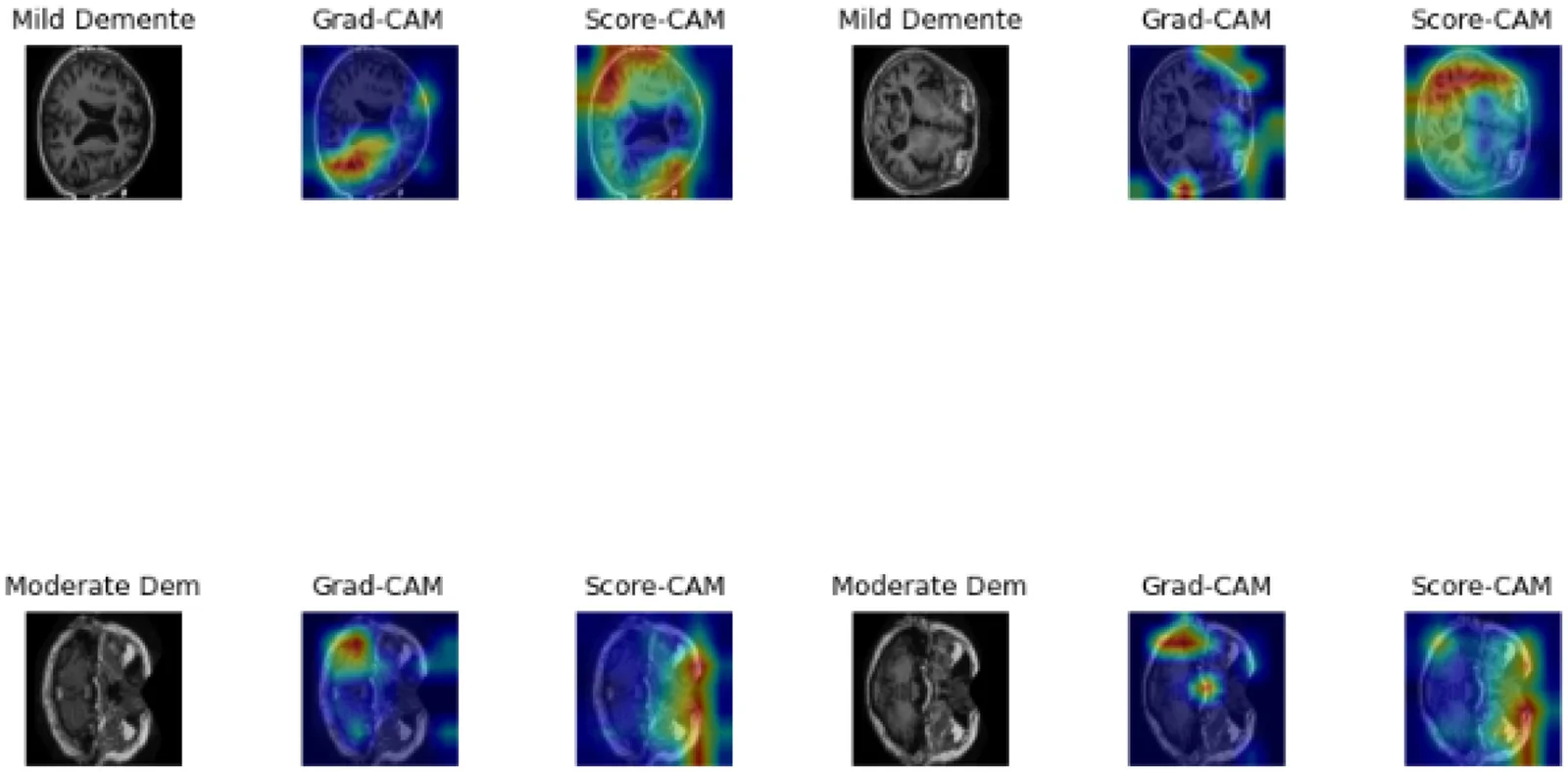
Representative post-hoc interpretability overlays produced with Grad-CAM (middle column of each pair) and Score-CAM (right column of each pair) on individual held-out MRI slices, shown here for two of the four Alzheimer's stages. Both methods concentrate attention on the cortical rim and adjacent parenchymal regions rather than on non-cerebral areas, and produce broadly consistent maps; Score-CAM distributes attention more broadly by aggregating over the top 24 channels of the deepest ConvNeXt feature map, whereas Grad-CAM sharpens onto the locations whose gradients are largest for the target class.

### 4.10 External validation on an independent dataset

To probe whether the accuracy on OASIS transfers to a data distribution the model has never seen, we evaluated the trained ConvNeXt–KAN pipeline on an independent four-class Alzheimer's MRI collection distributed on Kaggle,^1^ which uses the same clinical labels as OASIS (*Non Demented, Very Mild Demented, Mild Demented*, and *Moderate Demented*) but is acquired under different imaging conditions and includes different subjects. The model was applied *as-is*, with no fine-tuning, no threshold recalibration, and no re-training, so that the numbers reported here measure only the out-of-distribution generalization of the classifier learned on OASIS. The same preprocessing pipeline and the same four-crop test-time augmentation used in the internal evaluation were applied to the external images. For a like-for-like comparison with Table 1, the external set was balanced to 73 images per class (292 images in total).

Table 7 summarizes the overall metrics. The model reaches a test-time-augmented accuracy of 0.6164 on the external set, with an identical balanced accuracy of 0.6164 (confirming that the four classes contribute equally by construction) and a macro-averaged F1 of 0.5654; because the support is uniform, the weighted F1 coincides with the macro value at 0.5654. Relative to the 94.5% obtained on the OASIS held-out set, this is a substantial drop of approximately 33 percentage points, in line with the magnitude of degradation that is repeatedly observed for MRI-based classifiers when they are transported across acquisition sites without any adaptation. Crucially, it is precisely this gap—between an in-distribution number that is competitive with the literature and an out-of-distribution number that is markedly lower—that motivates the caution against single-dataset claims of near-perfect Alzheimer's staging.

**Table 7:** Zero-shot external validation of ConvNeXt–KAN on the independent four-class Alzheimer's MRI dataset (Kaggle, Preet Pal Singh).

Metric	Value
Test accuracy (TTA)	0.6164
Balanced accuracy	0.6164
Macro-averaged F1	0.5654
Weighted F1	0.5654
Number of test images	292 (73 per class)

No fine-tuning is applied; test-time augmentation matches the internal evaluation.

The class-wise picture is more informative than the aggregate. Figures 21, 22 show the external confusion matrices in absolute counts and row-normalized form, respectively. Three patterns are worth highlighting. First, the moderate dementia class continues to be recovered perfectly (73/73, 100% recall), replicating the internal-test behavior and consistent with the fact that moderate atrophy produces the most visually distinctive MRI signature and is therefore the least sensitive to acquisition-related domain shift. Second, non-demented recall drops only modestly (79.45% external vs. 82.19% internal), suggesting that the model's notion of a “normal-appearing” brain also generalizes reasonably well. Third, and by contrast, the two clinically intermediate stages absorb almost all of the additional error: mild dementia recall collapses from 98.63% to 10.96%, with the true mild-dementia scans redistributed mostly to very-mild-demented (43.84%) and moderate-demented (30.14%), and very-mild-demented recall falls from 97.26% to 56.16%, with the residual mass drifting toward non-demented (24.66%). This is consistent with the fact that the visual differences between adjacent stages are subtle even for expert raters, so that the model's decision boundary in this narrow region is the one most sensitive to a change in image intensity statistics, contrast, and slice selection between the two datasets.

**Figure 21:**
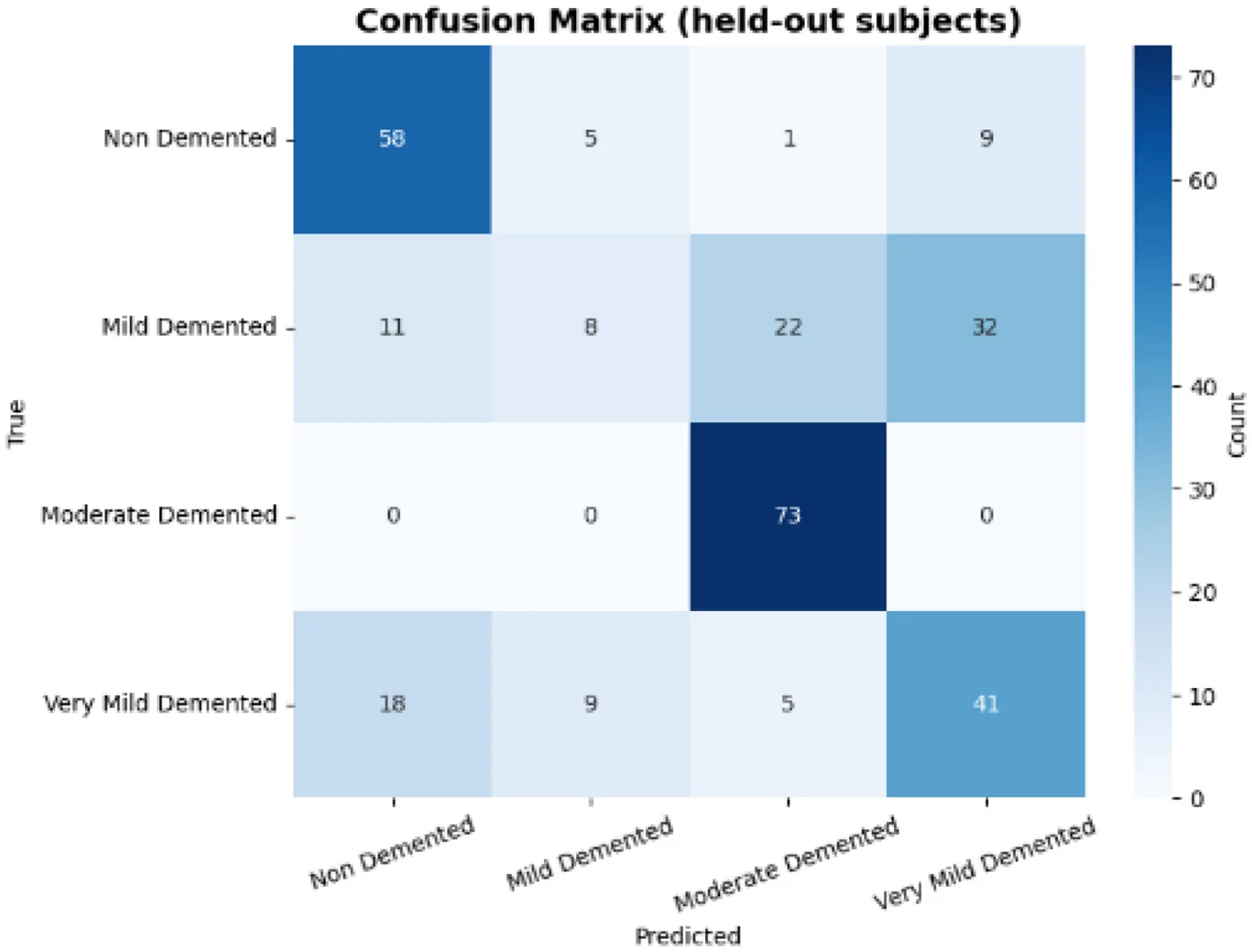
Confusion matrix (absolute counts) for zero-shot external validation of ConvNeXt–KAN on the independent four-class Alzheimer's MRI dataset (73 images per class, 292 total). Moderate dementia is again classified perfectly (73/73); most of the additional error concentrates on the two clinically intermediate stages, with mild-demented cases most frequently reassigned to very-mild-demented or moderate-demented.

**Figure 22:**
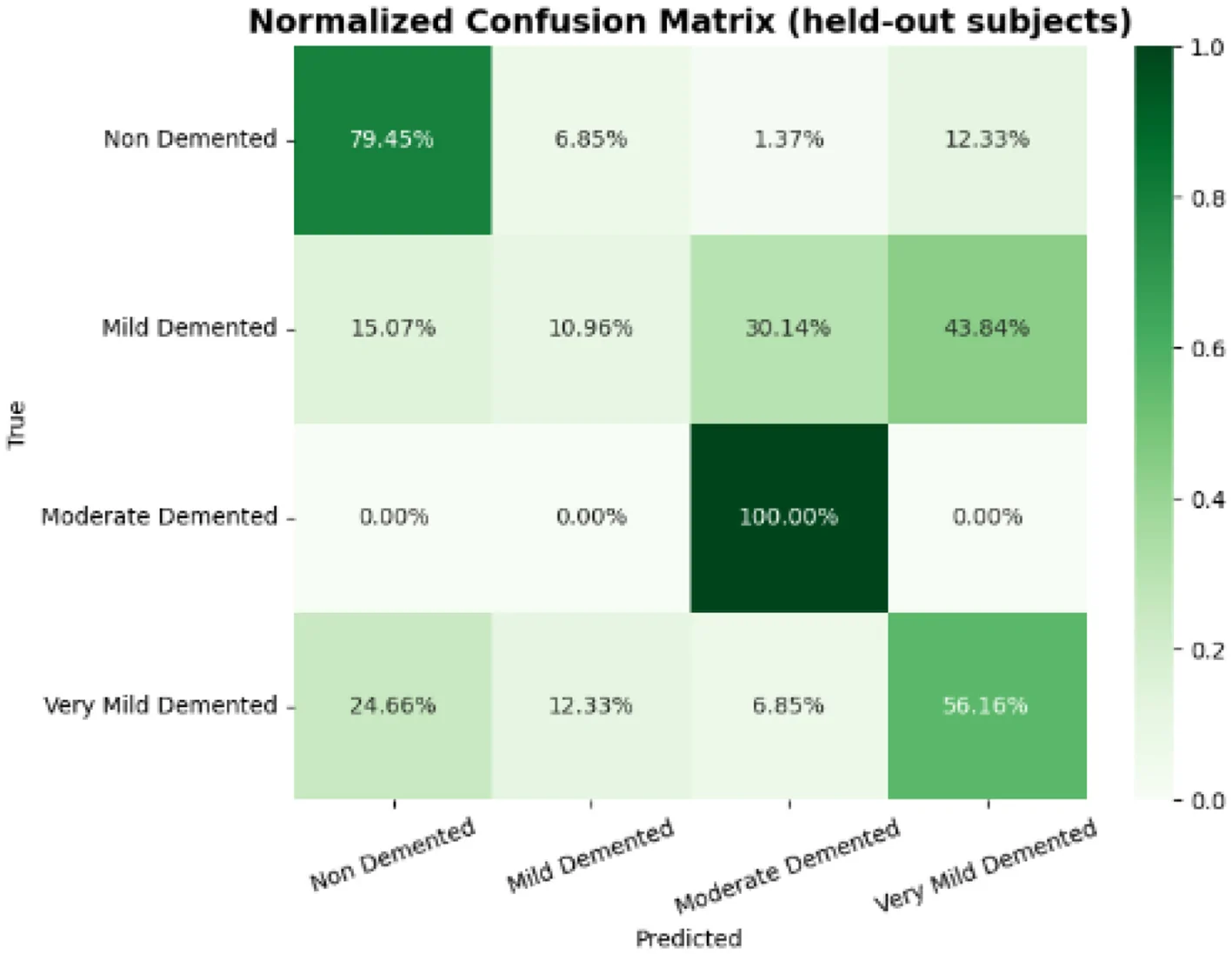
Row-normalized confusion matrix (recall per true class) on the external dataset. Moderate dementia is recovered with 100% recall and non-demented with 79.45%; the intermediate stages degrade more sharply, with mild-demented recall at 10.96% and very-mild-demented at 56.16%. The same qualitative pattern as the internal evaluation is preserved—errors cluster on adjacent stages—but the magnitude is larger, quantifying the cost of pure zero-shot transfer.

Taken together, the external evaluation both tempers the headline OASIS number and adds evidence for the article's main methodological argument. The gross ordering across classes—moderate, easiest, non-demented next, then very-mild, then mild—is preserved between the two datasets, which is non-trivial and suggests that the ConvNeXt–KAN features carry a genuine, if imperfect, signal about disease stage rather than a purely dataset-specific shortcut. At the same time, the collapse of the intermediate classes under zero-shot transfer indicates that any clinical deployment of a model of this kind would require domain adaptation (for example, a small amount of fine-tuning on the target site, or site-invariant training objectives) rather than direct reuse of the OASIS-trained weights; it reinforces the case for reporting external, multi-site numbers alongside the internal test in future studies.

### 4.11 Novelty and positioning relative to existing KAN-based medical imaging studies

To clarify the contribution of ConvNeXt–KAN relative to the growing body of KAN-based medical imaging research, Table 8 summarizes the key design differences between the proposed approach and the most closely related prior works. The central distinction is the *hybrid head design*: whereas MedKAN (Yang et al., 2025) builds a full KAN backbone with local and global information modules, and MedViTV2 (Manzari et al., 2026) replaces transformer feed-forward layers with KAN blocks throughout the network, our approach retains a pretrained convolutional feature extractor (ConvNeXt) and uses a KAN *only as the classification head*. This design exploits the strong transfer-learned representations of ConvNeXt, while introducing learnable-activation decision boundaries at minimal additional cost—the KAN head adds only 2.84% to total parameters and negligible inference latency (Section 4.8). Furthermore, to our knowledge, no prior KAN-based study has addressed the four-class OASIS Alzheimer's staging benchmark with an explicit imbalance-aware protocol (duplication-leakage control, class-capped oversampling, balanced test set) or reported zero-shot external validation on an independent dataset, both of which are contributions of this work.

**Table 8:** Comparison of ConvNeXt–KAN with existing KAN-based medical imaging studies.

Study	KAN scope	Backbone	Task	Dataset	Imbalance control	External validation
Liu et al. (2024)	Full backbone	KAN only	Toy/scientific	Various	No	No
Yang et al. (2025)	Full backbone	LIK+GIK	Multi-disease clf	MedMNIST	No	No
Bodner et al. (2024)	Conv layers	ConvKAN	General vision	MNIST etc.	No	No
Manzari et al. (2026)	Transformer FF	MedViT	Multi-disease clf	27 sets	Partial	No
Gao et al. (2025)	Multi-scale	CNN+KAN	Medical clf	Various	No	No
**Ours (ConvNeXt–KAN)**	**Head only**	**ConvNeXt**	**4-class AD staging**	**OASIS+Kaggle**	**Yes**	**Yes**

“Head only” means the KAN replaces only the final classifier; “Full backbone” means KAN layers permeate the feature extractor. Bold indicates the proposed method (ConvNeXt–KAN).

### 4.12 Discussion

The results support the central hypothesis: a learnable-activation KAN head on a transfer-learned ConvNeXt backbone yields accurate four-way Alzheimer's staging, with the largest residual error on the non-demented/very-mild boundary that is also the most difficult for clinicians.

The additional ablations in Sections 4.5 and 4.7–4.9, and the backbone comparison and novelty analysis in Sections 4.6 and 4.11 clarify where the model's headline number comes from and where it does not. The head-only comparison (Section 4.5) shows that under a shortened matched protocol, the KAN and MLP heads reach statistically indistinguishable accuracy on the internal test set (McNemar *p* = 0.52); the KAN head is favored on the threshold-independent ROC–AUC, while the MLP head is favored on threshold-dependent metrics, and the two heads trade a small margin. This finding calibrates the case for the KAN head: at the scale and epoch budget tested it is not empirically dominant, so the argument for adopting it rests on its learnable-activation expressive capacity and its negligible runtime cost (Section 4.8)—namely a 2.84% increase in total parameters, an essentially unchanged inference latency, and no measurable increase in wall-clock training time—rather than on a clear accuracy improvement in this regime. The imbalance-handling comparison in Section 4.7 is more decisive: image-space resampling on the training partition dominates focal loss, class-balanced cross-entropy, and feature-space SMOTE across accuracy, macro-F1, MCC, and kappa, with the SMOTE and focal-loss differences being statistically significant (McNemar *p* = 0.0002 and *p* = 0.039 respectively). Together with the leakage-controlled evaluation protocol, this supports our choice of resampling as the imbalance strategy for the primary run. The post-hoc saliency analysis in Section 4.9 shows that Grad-CAM and Score-CAM concentrate attention on the cortical rim and adjacent parenchymal regions rather than on non-cerebral areas, which is the qualitative pattern that would be expected if the model were relying on genuinely disease-relevant morphology.

Four limitations temper the headline number. First, although our protocol removes image-duplication leakage by splitting before oversampling and balancing the test set, the publicly distributed OASIS slices are not grouped by subject, so slices from one participant may still fall in different partitions; this can inflate accuracy and is a plausible explanation for the very high (often ≥99%) figures reported elsewhere on the same data. Genuinely patient-independent evaluation, therefore, requires a subject-level split, which we identify as the most important next step. Second, the external evaluation reported in Section 4.10 shows that zero-shot transfer to an independent four-class Alzheimer's MRI collection drops overall accuracy from 94.5% to 61.6%, with the intermediate stages carrying most of the additional error. This is not a failure of the architecture—the moderate class is still classified perfectly, and the ordering of class difficulty is preserved—but it is a clear signal that any clinical use would require domain adaptation to the target site rather than direct reuse of the OASIS-trained weights, and that broader multi-site validation remains a prerequisite for clinical claims.

Third, the present study employs a single train/validation/test split rather than *k*-fold cross-validation. This is a deliberate, practical choice: the extreme class imbalance in OASIS (moderate-dementia slices account for only 0.6% of the corpus) means that constructing *k* balanced, non-overlapping folds of sufficient size for the minority class is not feasible without heavy oversampling *inside* each fold, which in turn reintroduces the leakage concern that the evaluation protocol is designed to eliminate. Repeated hold-out evaluation (re-running the split with different random seeds) would address variance estimation without this complication, and is an important direction for future work. We also note that all reviewer-requested head comparisons, backbone comparisons, and imbalance-handling comparisons in this manuscript are conducted under a consistent, matched protocol on the same fixed test set, so the *relative* conclusions are unaffected by the use of a single split, even if the absolute numbers carry some variance. Cross-validated estimates of the primary model's accuracy and macro-F1, obtained once a subject-level split becomes feasible (i.e., when per-slice subject IDs are available), will be an important deliverable for the follow-up study.

Fourth, the external validation uses an independent Alzheimer's MRI collection distributed on Kaggle rather than the Alzheimer's Disease Neuroimaging Initiative (ADNI) dataset that is commonly recommended in the literature. ADNI access requires an application and data-use agreement, which was not in place during the preparation of this manuscript; the Kaggle collection was therefore used as the nearest available independent four-class benchmark. We fully concur that ADNI validation is a scientifically important step and include it explicitly in our future work plan. The external evaluation we do report nonetheless serves its intended purpose—it demonstrates measurable domain shift under zero-shot transfer and argues for caution against single-dataset claims—and the Kaggle collection uses the same four-class label set and was acquired from different subjects under different imaging conditions, making it a meaningful, if imperfect, proxy for out-of-distribution generalization.

## 5 Conclusion

We presented ConvNeXt–KAN, a model that combines a transfer-learned ConvNeXt backbone with a Kolmogorov–Arnold Network classification head—an architecture in which the head's activations are learned rather than fixed—for multi-class staging of Alzheimer's disease from brain MRI. The KAN head, implemented efficiently with Gaussian radial basis functions, is designed to capture the subtle non-linear texture and atrophy patterns that conventional MLP heads model poorly, and it is trained with a robustness-oriented recipe (MixUp and CutMix, label smoothing, AdamW, a cosine schedule with warm-up, and test-time augmentation) together with a data strategy that exploits far more of the imbalanced OASIS collection while evaluating on a fixed, balanced, leakage-controlled test set. The model achieved 94.5% accuracy, a macro-F1 of 0.944, and a mean ROC–AUC of 0.991 on the internal test set, and an ablation confirmed that pretrained features and expanded data are the principal contributors. A zero-shot evaluation on an independent four-class Alzheimer's MRI collection reached 61.6% accuracy and a macro-F1 of 0.565 without any fine-tuning, with moderate dementia still recovered perfectly and the intermediate stages carrying most of the domain-shift error—evidence that the learned features generalize partially, but that domain adaptation would be required for clinical reuse of the OASIS-trained weights. Beyond the numbers, the study contributes a transparent treatment of data utilization and evaluation, including an explicit account of subject-level leakage as a confound in prior work and a quantified external comparison. A backbone comparison (Section 4.6) confirms that ConvNeXt-Tiny offers the best capacity–regularization trade-off in this data regime: ConvNeXt-Base provides a small gain (+0.017 accuracy) at three times the parameter count, while EfficientNetB4, DenseNet121, and ViT-B/16 all trail ConvNeXt-Tiny under matched conditions. A comparative novelty table (Section 4.11) positions ConvNeXt–KAN within the growing body of KAN-based medical imaging literature and highlights the unique combination of hybrid head design, imbalance-aware protocol, and external validation that distinguishes this work. Future work will (i) adopt a subject-level split for patient-independent evaluation and use repeated *k*-fold cross-validation once subject-level grouping information is available; (ii) validate on ADNI and other multi-site clinical cohorts to establish genuine cross-site generalizability; (iii) explore ConvNeXt-Base as the backbone with domain-adaptation objectives; (iv) add probability calibration and additional gradient-based explanation to improve clinical trust; and (v) investigate quantitative localization faithfulness metrics (deletion/insertion curves, ROI-based analysis) for a more rigorous assessment of model interpretability.

## Data Availability

The datasets analysed in this study are publicly available. The cross-sectional OASIS MRI collection is available from the Open Access Series of Imaging Studies (https://www.oasis-brains.org) and in the four-class slice-level form distributed on Kaggle. The independent dataset used for external validation is available at https://www.kaggle.com/datasets/preetpalsingh25/alzheimers-dataset-4-class-of-images. All other original contributions presented in the study are included in the article; further inquiries can be directed to the corresponding author.

## Footnotes

1 Preet Pal Singh, “Alzheimer's Dataset (4 class of Images),” Kaggle. Available at https://www.kaggle.com/datasets/preetpalsingh25/alzheimers-dataset-4-class-of-images. Accessed [date] for external validation in this study.
